# The Importance of Sex-Based Comparisons in Preclinical Nanomedicine and Regenerative Chronic Wound Therapies

**DOI:** 10.1021/acsbiomaterials.5c00996

**Published:** 2025-09-26

**Authors:** Negar Mahmoudi, Mohsin Hassan Saeed, Lars Peereboom, Xinyue Liu, David R. Nisbet, Morteza Mahmoudi

**Affiliations:** † The Graeme Clark Institute, 2219 The University of Melbourne, Melbourne VIC 3010, Australia; ‡ Department of Biomedical Engineering, Faculty of Engineering and Information Technology, The University of Melbourne, Melbourne VIC 3010, Australia; § Department of Chemical Engineering and Materials Science, 3078 Michigan State University, East Lansing, Michigan 48824, United States; ∥ Melbourne Medical School, Faculty of Medicine, Dentistry and Health Science, The University of Melbourne, Melbourne VIC 3010, Australia; ⊥ Precision Health Program, 3078 Michigan State University, East Lansing, Michigan 48824, United States; # Department of Radiology, College of Human Medicine, Michigan State University, East Lansing, Michigan 48824, United States; ∇ Connors Center for Women’s Health & Gender Biology, Brigham & Women’s Hospital, Harvard Medical School, Boston, Massachusetts 02115, United States

**Keywords:** chronic wounds, nanomedicine, regenerative medicine, biomaterials, sex-specific responses, animal models

## Abstract

It is increasingly recognized that immune responses, plasma proteomes, and various biosystem reactions to advanced therapeutics, such as nanomedicine and regenerative medicine, differ by gender. While sex is a crucial factor influencing the safety and efficacy of these therapies, there is a notable lack of robust consideration of sex differences in both preclinical and clinical studies. This Perspective examines the current literature on the role of sex in nanomedicine and biomaterial-based products for preclinical chronic wound healing. While there is a growing trend of using both male and female animal models, most studies lack direct, side-by-side comparisons of sex-specific outcomes. Specifically, 77.8% of nanomedicine and 85.3% of biomaterial studies exclusively employed either male or female animals. Only 7.4% of nanomedicine and 2.1% of biomaterials publications included both sexes; however, apart from two studies, most did not perform direct sex-based comparisons. Instead, they often assigned different sexes to separate species, wound types, or experimental conditions (e.g., noninfected versus infected wounds). To deepen our understanding of sex differences in advanced therapeutics, future research should focus on direct, side-by-side comparisons of male and female *in vivo* models for safety and efficacy.

## Graphical abstract

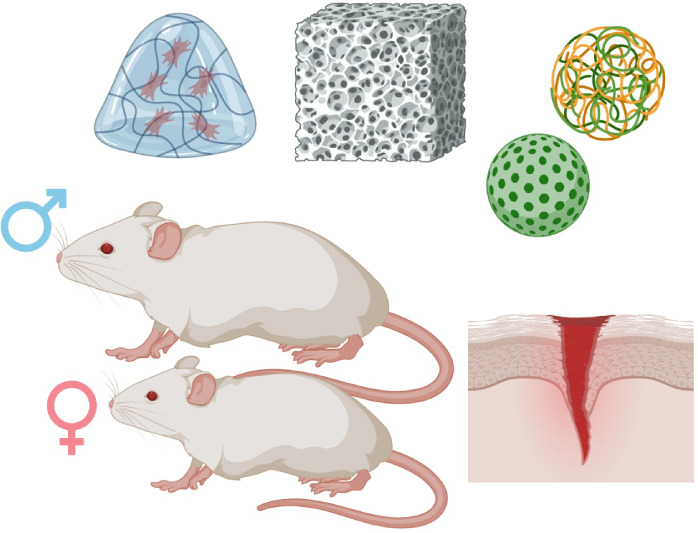

## Introduction

The skin serves as a vital, static barrier that protects the body against myriad diseases and contaminants.^1^ Annually, millions of individuals suffer from impaired wound healing, leading to increased mortality rates and significant burdens on healthcare systems.^2^ Wounds resulting from surgery, burns, or chronic diseases can cause skin damage, making infections more likely. While acute wounds typically heal efficiently, chronic wounds present complex challenges that impede the healing process. A hostile microenvironment within the wound bed, characterized by poor cell migration and proliferation, inadequate angiogenesis, persistent microbial infection, and excessive inflammation, contributes to prolonged and complicated healing.^3^

## Overview of Acute Wound Healing Stages

Acute wound healing is the body’s natural and timely response to injury, involving a well-coordinated series of physiological processes aimed at restoring the integrity of the cutaneous barrier and underlying tissues. This process is characterized by four sequential and overlapping phases:^4^ hemostasis, inflammation, proliferation, and remodeling.

### Hemostasis

Hemostasis is the initial response to injury, with the main goal of stopping bleeding and minimizing hemorrhage. This phase involves vasoconstriction, platelet aggregation, and clot formation.^5^ Vasoconstriction, triggered by vasoconstrictors like endothelin, temporarily reduces blood flow. Platelets then aggregate, adhering to exposed collagen in the extracellular matrix, and secrete adhesive glycoproteins to form a platelet plug.^5^ This is followed by secondary hemostasis, where the clot stabilizes through fibrin matrix deposition, serving as a scaffold for the incoming cells involved in subsequent healing phases.

### Inflammation

This phase begins immediately after injury and lasts up to 6 days, following vasoconstriction and platelet degranulation, which release histamine and other mediators.^6,7^ This leads to vasodilation and increased vascular permeability, allowing for the influx of inflammatory cells like neutrophils and monocytes. Neutrophils perform phagocytosis to remove debris and pathogens, while monocytes differentiate into macrophages.^4,5^ To prevent prolonged inflammation, macrophages secrete cytokines that regulate inflammation, stimulate angiogenesis, and promote the formation of granulation tissue.

### Proliferation

Starting around day four and lasting up to 21 days, this phase is marked by granulation tissue formation, wound contraction, and angiogenesis.^6^ Angiogenesis is driven by growth factors such as transforming growth factor beta (TGF-β), platelet-derived growth factor (PDGF), fibroblast growth factor (FGF), and vascular endothelial growth factor (VEGF).^8^ Fibroblasts migrate to the wound site, laying down extracellular matrix components like hyaluronan and collagen and forming granulation tissue. This tissue supports re-epithelialization, where keratinocytes migrate to cover the wound, and myofibroblasts facilitate wound contraction by drawing the wound edges together.

### Remodeling

Beginning around week three and potentially lasting for a year or more, this phase involves the replacement of type III collagen with type I collagen, increasing tensile strength up to 80% of normal skin. Fibroblasts continue to remodel the extracellular matrix, depositing and degrading collagen to form organized, cross-linked networks.^8^ The maturation of scar tissue reduces the vascularity, resulting in a stable, mature scar. Effective progression through each phase is crucial for successful wound healing, with any disruption potentially leading to chronic wounds.^9^

## Key Differences Between Acute and Chronic Wound Healing

The key differences between acute and chronic wound healing lie in the duration, underlying causes, and efficiency of the healing process, together with healing complications.^10^

### Duration

Acute wounds typically heal within a predictable and short time frame, usually weeks to a few months, depending on the severity and size of the wound. Chronic wounds, however, persist for a prolonged period, often defined as wounds that do not heal within 3 months and may linger for years without proper treatment.^11^

### Underlying Causes

Acute wounds are often caused by trauma (e.g., cuts, abrasions, and surgical incisions), burns, or other sudden injuries. Chronic wounds are frequently associated with underlying health conditions such as diabetes, venous insufficiency, arterial disease, pressure ulcers, and other systemic issues that impair circulation, sensation, or immune function.^11^

### Healing Efficiency

In acute wound healing, the body’s repair mechanisms function efficiently, leading to timely closure and restoration of tissue integrity. The wound environment is typically optimal for healing. In chronic wounds, the body’s repair mechanisms are compromised, often getting stuck in one of the healing phases, most commonly the inflammatory phase, due to various underlying factors that impair the normal progression of healing.^12^

### Complications

Complications are less common in acute wounds and, when present, are usually due to external factors such as infection or repeated trauma. Chronic wounds are more prone to complications, including persistent infection with resistant bacteria, biofilm formation, and increased risk of severe consequences such as bone infection or sepsis.^13^

## Biomaterials and Regenerative Medicine in Chronic Wound Healing

Recent advances in nanomedicine and biomaterials have shown promising potential to enhance wound healing, particularly in chronic cases.^14–18^ These innovative strategies interact with the wound microenvironment to stimulate cellular and molecular processes, including pathogen inhibition,^19–22^ inflammation modulation,^23,24^ immune regulation,^25–27^ and angiogenesis^28–30^ promotion. By harnessing these biological responses, nanomedicine and biomaterials can transform nonhealing environments into ones conducive to tissue repair and regeneration.

In numerous experimental studies, wound dressings, including drug-loaded polymeric micro- and nanospheres, nanoparticles (NPs), nanofibrous structures, scaffolds, cryogels, and hydrogels, were used to enhance wound healing. In brief, metal NPs,^19^ lipid NPs,^31–33^ and ultrasmall NPs^34^ are used as wound dressings and as delivery systems for chemical substances (e.g., active drugs, antibiotics, growth factors, and other therapeutic agents) over an extended period, thereby accelerating the wound healing process. Moreover, several natural polymers (e.g., chitosan,^35^ gelatin,^36^ dextran,^37^ hyaluronic acid,^38^ collagen, and alginate^39^) and synthetic polymers (e.g., poly­(ethylene glycol) (PEG),^40,41^ polyurethane (PU),^42,43^ and poly­(vinyl alcohol) (PVA)^44–46^ ), nanofibrous membranes either alone or as hybrid membranes (e.g., poly­(lactide-*co*-glycolic acid) (PLGA)/aloe vera (AV)^47^ extract, silk fibroin (SF)/PLGA, and polycaprolactone (PCL)^48^), composite patches (e.g., chitosan/collagen/chondroitin sulfate/elastin/hyaluronic acid),^28^ self-healing hydrogels (carboxymethyl chitosan (CMCS)-protocatechualdehyde (PCA)^49^), bioglass composite hydrogels,^50^ and 3D printed hydrogels (e.g., VEGF-coated tetrapodal zinc oxide (t-ZnO)-laden GelMA,^27^ bovine serum albumin (BSA), and AV gel^51^) have been fabricated as multifaceted wound dressings through various chemical or physical cross-links.

## Sex Affects the Healing Process in Chronic Wounds

The importance of considering sex as a biological variable in wound healing has gained increasing recognition since the early 2000s.^52,53^ Wound healing disparities between male and female patients are evident at multiple physiological levels due to differences in tissue properties (e.g., skin stiffness, skin prestrain, sebum production),^54–56^ wound exudate compositions (e.g., growth factors, hormones, cytokines), immune system functions, and injury responses^57–63^ (Figure 1). For example, at the tissue level, men’s generally stiffer and thicker skin creates elevated tension at wound edges, making it more resistant to common wound closure techniques compared to the softer, thinner skin of women.^57,64^ At the cellular level, sex differences in wound exudates’ compositions can alter the formation of protein coronas around nanomedicines delivered from active skin patches, affecting their uptake and activity.^65,66^ Sex also impacts immune system reactions in the wound microenvironment, which influence healing outcomes. For example, animal studies suggest that female mice have more wound macrophages than males,^67^ which correlates with slower healing in diabetic and chronic venous leg ulcers.^67^ While estrogen speeds wound healing,^68^ high macrophage counts in women can delay it, revealing that the outcome depends on a balance of sex-dependent factors. While female patients tend to have more M2 macrophages,^68^ which are associated with anti-inflammatory responses, males typically exhibit higher levels of M1 macrophages, which are linked to pro-inflammatory reactions.^69^ This may be explained by androgen crosstalk with macrophages. Chronic conditions and comorbidities such as poor circulation^70^ further complicate sex-specific roles of macrophages. Therefore, incorporating sex differences into wound healing research is key to developing personalized treatments by addressing how sex impacts immunity, tissue repair, and recurrence, leading to better outcomes.

**Figure 1:**
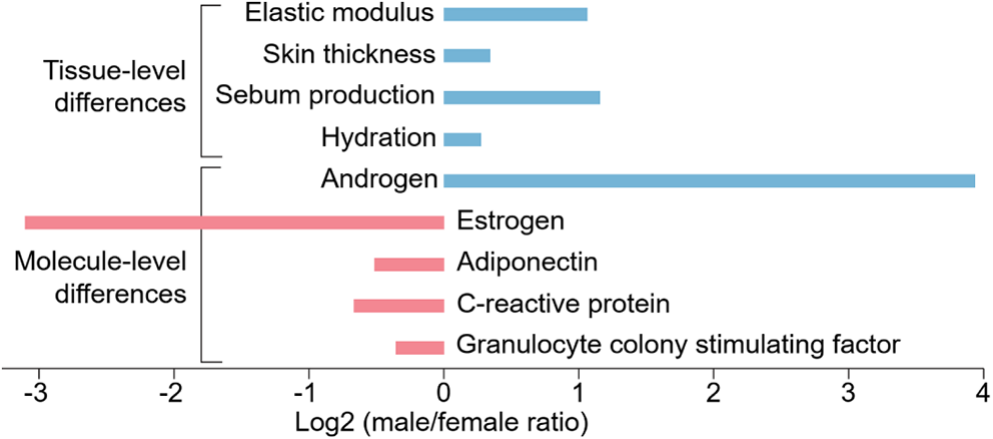
Tissue- and molecule-level differences related to wound healing.

## What Is Covered in This Perspective?

In our previous publications,^65,71,72^ we have extensively investigated the mechanisms underlying sex-specific differences in wound healing, emphasizing how biological sex influences cellular responses, immune function, and tissue regeneration. Building on this foundation, we have also analyzed the landscape of nanomedicine and biomaterials currently in clinical development for treating diabetic foot ulcers and venous leg ulcers, with particular attention to the representation of male and female participants in these clinical trials.^72^ Recognizing that sex-specific factors can significantly shape the biological identity of nanomedicine formulations,^66^ which affect their interactions with biosystems, we have explored how these variables influence responses in biosystems across different biological contexts.

This perspective aims to critically assess the current state of knowledge regarding sex-specific data availability in the preclinical stage. Specifically, we focus on evaluating how and to what extent sex differences are documented within the scientific literature, with a particular emphasis on research involving animal models. Understanding these differences is crucial for translating findings from preclinical studies to human applications, as animal studies often form the basis for safety and efficacy assessments.

## Literature Analysis on Considering Sex in Preclinical Models

Recently, we analyzed the sex distribution of participants in key clinical trials involving advanced bioengineered and nanomedicine products (Table 1) for lower extremity ulcers, such as diabetic foot ulcers and venous leg ulcers.^72^ Our review revealed notable sex differences in recruitment; specifically, females were underrepresented compared to their prevalence in the general lower extremity ulcer patient population. There was a significant over-representation of males in the intervention groups, which poses challenges for accurately assessing the efficacy and safety of these therapies in females. Furthermore, only three out of 25 studies that incorporated sex analysis specifically evaluated sex-based differences in outcomes regarding safety or efficacy.

**Table 1:** Examples of Bioengineered and Nanomedicine-Based Wound Dressing Products Currently in Clinical Trials, Primarily Used for Diabetic Foot Ulcers and Venous Leg Ulcers^a^

Product Name	Company
HP802-247	Smith and Nephew, Inc., Memphis, TN, USA
OASIS Extracellular Matrix	Smith and Nephew, Inc., Memphis, TN, USA
Becaplermin gel (Regranex)	Smith and Nephew, Inc., Memphis, TN, USA
LL-37	Pergamum AB, Stockholm, Sweden
Apligraf	Organogenesis, Inc., Canton, MA, USA
Dermagraft	Organogenesis, Inc., Canton, MA, USA
Viscopaste	Smith and Nephew, Inc., Memphis, TN, USA
Acoband	Auspharm, Balcatta, Australia
Kaltostat	Faulding Pharmaceuticals, Adelaide, Australia
Biatain and Biatain-Ag	Coloplast A/S, Humlebæk, Denmark
RECELL	AVITA Medical, Valencia, CA
Octenilin wound gel	Schuelke and Mayr, Norderstedt, Germany
Aquacel Ag	Aquacel Ag: Convatec, Skillman, New Jersey, USA
Acticoat, Acticoat 7, Acticoat Absorbent	Acticoat: Smith and Nephew, London, UK
Contreet Foam	Contreet Foam: Coloplast, Humlebæk, Denmark
Urgotul SSD	Urgotul SSD: Laboratoires Urgo, Chenôve, France
Derma-Gide	Geistlich Pharma North America, Inc., Princeton, NJ, USA
Integra Flowable Wound Matrix	Integra LifeScience, Corp., Princeton, NJ, USA
Omnigraft Dermal Regeneration Matrix	Integra LifeScience, Corp., Princeton, NJ, USA
UrgoStart Contact	Laboratoires Urgo Medical, Chenôve, France
Apligraf	Organogenesis, Inc., Canton, MA, USA
EpiFix	MIMEDX Group, Inc., Marietta, GA, USA
LeucoPatch	Reapplix ApS, Birkerød, Denmark

a The information presented in this table is sourced from ref. 72, with permission from Oxford University Press.

To further investigate this critical issue, we systematically examined how sex is considered in preclinical assessments of nanomedicine and biomaterials specifically designed for chronic wound therapy. We conducted a thorough review of the literature, analyzing the sex distribution of animal models used in relevant studies, which is summarized in Tables 2 and 3. Our review revealed a significant gap: the majority of preclinical research predominantly utilizes male animals, with females either underrepresented or entirely absent in many studies (see Figure 2a for nanomedicine and Figure 2b for bioengineered materials). Furthermore, among studies that included both males and females, direct comparisons of sex-specific responses were rare. Few studies involving both sexes were able to compare sex-specific responses (only two studies showed sex-based differences in their findings; for example, males healed faster with wound dressings and responded better to antibiotics than females^73^). However, in most cases, researchers assigned each sex to distinct experimental variables, such as different species, wound types, or specific pathological conditions, rather than conducting direct sex-based comparisons within the same experimental framework.

**Figure 2:**
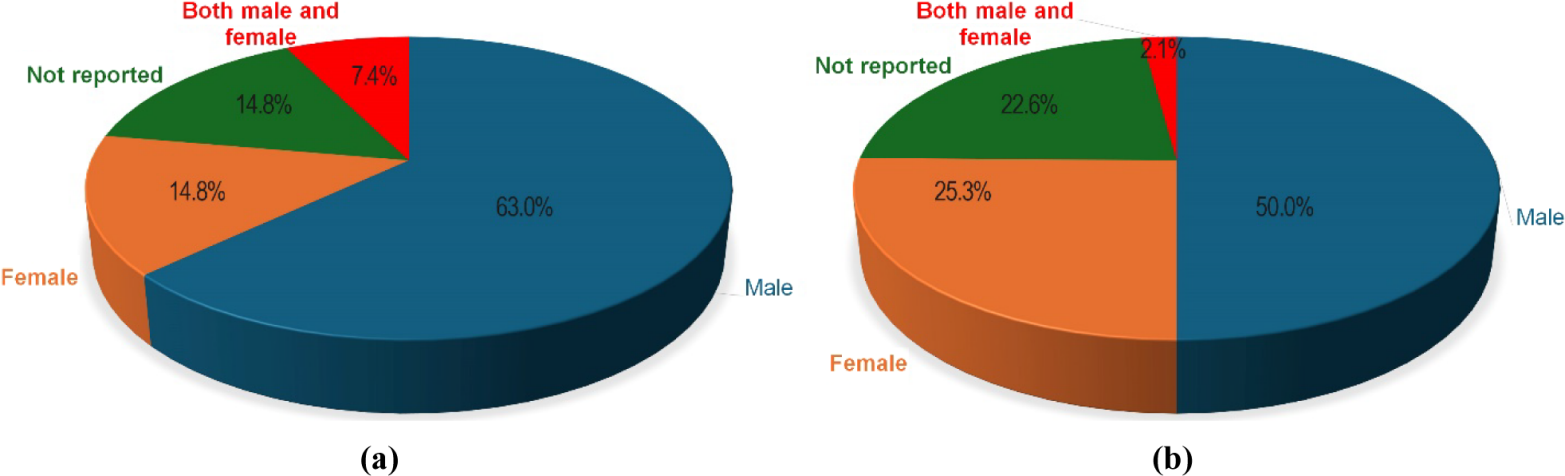
Distribution of the sex of the employed animals in chronic wound studies using bioengineered materials and nanomedicine products. (a,)­(b): We conducted a literature review on the distribution of animal sexes in studies focusing on nanomedicine for chronic wound healing, utilizing PubMed on March 5, 2024, with the following keywords: (a) “chronic”, “wound healing”, and “nanomedicine”, and (b) “chronic”, “wound healing”, and “biomaterials”. The search yielded (a) 134 and (b) 624 papers, from which we selected (a) 54 and (b) 189 that involved *in vivo* analyses. Most of these studies [(b):a) 77.8% and (b) 85.3%] exclusively utilized either male or female animals. Among the studies, only (a) 7.4% and (b) 2.1% employed both female and male animals; however, none of these studies undertook a comparison of sex-specific responses [except for two studies in (b)]. Instead, each sex was assigned to different species, wound types, or specific conditions; for instance, one sex might be used for studies on noninfected wounds while the other for infected wounds.

**Table 2:** Distribution of Animal Sexes and Major Outcomes of the Studies Focusing on Nanomedicine for Chronic Wound Healing

Chronic wound type	Nanomedicine	Animal	Sex	Outcome	REF
**Diabetic (Type I) Mode**	Laponite containing platelet-rich plasma (PRP)-derived gel (PLDG) loaded with deferoxamine (DFO)	Sprague–Dawley (SD) rats	Male	Enhanced sustained release of growth factors	^76^
A Full-Thickness Wound (8 mm in Diameter) Using a Punch Biopsy				Elevated levels of M2 macrophages	
				Accelerated healing of diabetic wound	
**Inflammation Model**	Rutin loaded into zeolitic imidazolate framework-8 (ZIF-8)	BALB/c mice	Male	Exhibited anti-inflammatory and bactericidal effects	^19^
				Demonstrated antioxidant properties and facilitated macrophage polarization	
				Minimized tissue damage and oxidative stress through the scavenging of excessive reactive oxygen species (ROS) by zinc ions (Zn^2+^)	
**Diabetic Mice Wound Model**	Hydrogel consisting of alginate (at 2.5% oxidation degree) and calcium-activated PRP	db/db (BKS.Cg-m+/+ Lepr^db^/J) mice	Male	Enhanced cell adhesion and proliferation without cytotoxicity (*in vitro* on human fibroblasts and keratinocytes)	^39^
A Full-Thickness Wound (8 mm in Diameter) Using a Punch Biopsy	PRP-HG-2.5%			No significant differences observed *in vivo*	
**Diabetic (Type I)**	Iontophoretic antibiotic (vancomycin/daptomycin) delivery using high-intensity DC electric current-based biofilm treatment system	BALB/c Homozygous B6.BKS(D)-Lepr^db^/J mouse	N/A	Decreased bacterial count from 10^9.0^ CFU g^–1^ to 104.6 CFU g^–1^ at 1-day post-treatment and to 10^3.3^ CFU g^–1^ at 7-day post-treatment	^20^
Mouse-Based Skin Wound Model Infected with the MRSA Biofilm					
**Diabetic Mice**	Topical administration of recombinant human epidermal growth factor (rhEGF)-loaded lipid nanoparticles	db/db (BKS.Cg-m+/+Lepr^db^/J) mice	Male	Enhanced wound healing evidenced by wound closure, normalization of the inflammatory response, and re-epithelialization	^31^
A Full-Thickness Wound (8 mm in Diameter) Using a Punch Biopsy	Solid lipid nanoparticles (SLN)-rhEGF and nanostructured lipid carries (NLC)-rhEGF				
**Diabetic Mice**	Soy protein isolate (SPI)/β-chitin sponge-like scaffolds (SLS) laden with human mesenchymal stromal cells from hair follicle or adipose tissue	db/db (BKS.Cg-m+/+Lepr^db^/J) mice	Male	Enhanced wound closure and re-epithelialization	^77^
Two Full-Thickness Wound (8 mm in Diameter) Using a Punch Biopsy				Reduced ROS and hyperglycemia	
				Promoted collagen deposition and neovascularization	
				Reduced inflammation	
Wound Size (6 cm × 5 cm with 2 mm in Depth with the Subcutaneous Fat Left) and Position (2 cm Between Wounds)	Topical administration of rhEGF-loaded lipid nanoparticles	White pigs	Female	Accelerated wound closure	^32^
				Enhanced fibroblast migration and proliferation	
				Enhanced collagen deposition	
				Reduced inflammatory response	
**Diabetic Mice**	PLGA nanofibrous membrane containing (rhEGF) and Aloe vera (AV) extract. “PLGA-AV-EGF nanofibrous membrane”	db/db (BKS.Cg-m +/+ Lepr^db^/J) mice	Male	Promoted wound closure and re-epithelialization	^47^
A Full-Thickness Wound (8 mm in Diameter) Using a Punch Biopsy				Effectively suppressed bacterial proliferation	
**Diabetic Mice**	Composite polyvinyl alcohol (PVA)/chitosan electrosupn nanofibers as a delivery platform to release tea tree oil nanoliposomes (TTO-NLs) (TTO-NL@PCS)	BKS-Lepr^em2Cd479^/Gpt mice	Male	Enhanced prostaglandin F2α (PGF2α) and FP receptor signaling	^23^
Full-Layer Wounds (12 mm Diameter) Combined with MRSA Infection				Increased expression of interleukins IL-1β and IL-6	
				Diminished the inflammatory response	
				Elevated expression of vascular endothelial growth factor (VEGF) in the wound area	
**Ischemic Wounds**	Anti-miR-210 inhibitor (Exiqon) solution (AM-210) encapsulated in antihypoxamiR functionalized gramicidin lipid nanoparticles (AFGLN) (AFGLN_miR‑210_)	C57BL/6 mice	Male	Reduced levels of miR-210	^78^
Wound (3 mm in Diameter) Using a Bi-Pedicle Flap Model				Enhanced closure of ischemic wounds	
Four Equidistant Full-Thickness Excisional Wounds (4.5 mm in Diameter)	Antagomir-205 (inhibition/downregulation of miR-205)	C57BL mice	Male	Reactivated integrin alpha 5 (ITGA5) expression	^79^
				Encouraged keratinocyte migration	
A Full-Thickness Skin Wounds (10 mm in Diameter) on the Back	Apoptotic small extracellular vesicles (apoSEVs)-loaded gelatin methacryloyl (GelMA)	db/db (BKS-Dock Lepr^em2Cd479^) mice	Male	Enhanced wound healing	^25^
				Induced macrophage phenotypes polarization from M1 to M2	
				Supported collagen accumulation, angiogenesis, and the regulation of immune responses	
**Central Back Incisional Wound Model**	Indocyanine green (ICG)-mediated photo-dynamic therapy (PDT) ICG-PDT	C57BL/6 mice	Female	Enhanced wound healing	^80^
A Full-Thickness Skin Wound (20 mm)					
**Diabetic Mice**	Composite nanofibrous membranes of PLGA/Aloe vera containing lipid nanoparticles (NLCs)	db/db (BKS.Cg-m +/+ Lepr^db^/J) mice	Male	Enhanced wound healing evidenced by a significant decrease in wound size	^81^
A Full-Thickness Wound (8 mm in Diameter) Using a Punch Biopsy					
**Infected Diabetic Wound Model**	Oxidized carboxymethyl cellulose (OCMC)-tobramycin (Tob)/polyethylenimine (PEI injectable cationic hydrogel	C57BL/6 mice	Male	Demonstrated sustained antibacterial effectiveness	^21^
A Full-Thickness Wound (10 mm in Diameter) Dorsal Skin Punch Wounds				Enhanced wound closure rate	
**Tumor-Induced Wound and Diabetic Wound Model**	Injectable thermosensitive hydrogel with the integration of nanosized black titania (B-TiO_2–x_) nanoparticles into a chitosan (CTS) matrix. “BT-CTS thermo-gel”	Balb/c mice (tumor-induced model)	Male	Effectively suppressed tumor cell proliferation while simultaneously enhancing skin tissue regeneration	^82^
A Full-Thickness Wound (10 mm in Diameter) in Either Tumor Site or on the Skin		C57BL/6 mice (diabetic model)		Encouraged the growth of healthy skin cells	
**Polygenic, Moderate Type 2 Diabetic Infected Wound Model**	Gelatin/polyvinyl alcohol (PVA) electrospun nanofibers embedded with cephradine (antibiotic)	NONcNZO10/LtJ known as “NcZ10” mice	Male	Exhibited efficient bacterial clearance on day 11	^83^
A Full-Thickness (7 mm in Diameter) Using a Punch Biopsy				Enhanced chronic wound healing	
**Excisional Wound Splinting Model**	Asymmetric electrospun polycaprolactone (PCL) nanofibers decorated with electrosprayed poly(lactic-*co*-glycolic acid) microparticles (PLGA MPs) and loaded with thymol (THY)	SKH1 hairless mice	Male	Attenuated bacterial load in wound after 7 days	^22^
Two Full-Thickness (8 mm in Diameter) Using a Punch Biopsy					
**Genetically Diabetic Mice**	NPs consist of two chimeric fusion proteins comprising elastin-like peptides (ELPs) and either platelet-derived growth factor (PDGF) or a human neutrophil elastase (HNE) inhibiting peptide followed by suspension in fibrin gel	B6.BKS(D)-Lepr^db^/J) mice	Female	Significantly reduced inflammation	^24^
A Full-Thickness Excisional Square Wound (1 × 1 cm^2^) on the Upper Back of Each Mouse Using Sharp Scissors				Improved wound healing and collagen deposition	
**Diabetic Mice Model**	Topical hydrogel containing low-molecular-weight protamine (LMWP)-growth factor (GFs), quercetin (QCN)-NE, and oxygen-carrying 1-bromoperfluorooctane (OXY-PFOB-NE)	C57BL/6 mice	Female	Accelerated wound healing	^84^
A Full-Thickness Wound (8 mm in Diameter) at the Center of the Dorsal Skin Using a Punch Biopsy					
	(LMWP-GFs/QCN-NE/OXY-FPOB-NE-GEL)				
**CCl_4_-Induced Fibrotic Mice**	Rriociguat together with a tailor-designed galactose-PEGylated bilirubin nanomedicine (Sel@GBRNPs) with ROS scavenging and apoptosis inhibition properties	C57bl/6N mice	Female	Attenuated the stimulation of HSC activation and ECM deposition	^40^
**Glycerol-Induced Acute Kidney Injury (AKI) Model, and Diabetic Wound Healing Model**	Cu_5.4_O ultrasmall nanoparticles (USNPs)	BALB/c mice (glycerol-induced AKI model)	Female	Significant reduction (*p* < 0.001), of ALT and AST levels in Cu_5.4_O USNPs-treated AILI mice compared to AILI mice without treatment	^34^
A Full-Thickness (6 mm in Diameter) Using a Punch Biopsy at the Dorsal Skin		BALB/c mouse (diabetic wound healing)	Male	Faster diabetic wound healing rate in Cu_5.4_O USNPs group (accelerated wound healing process)	
		BALB/c mice (to evaluate the biocompatibility)	N/A	Alleviated renal injury through significant inhibition of MAPK signaling pathway and after Cu_5.4_O USNPs treatment	
		BALB/c mice (to evaluate the *in vivo* toxicity)	N/A	Significant upregulation of tissue repair related genes (e.g., fibroblast growth factor 10 (FGF10), hepatocyte growth factor (HGF), NOTCH1, and wingless-type MMTV integration site family member 7A (WNT7A)) after Cu_5.4_O USNPs treatment	
		BALB/c mice (to detect the accumulation of Cu_5.4_O USNPs in the major organs)	Female		
**Infected Diabetic Wound Model**	Drug-loaded composite cellulose acetate (CA) electrospun nanofibers, consisting of mixture of CA and poly(ethylene oxide) (PEO) nanofibers embedded with either methylene blue (MB) or ciprofloxacin (Cipro)	Swiss albino mice	Male	Elevated re-epithelialization	^85^
A Wound (1 × 1 cm^2^) on the Back of Dorsal Surface Using a Surgical Scalpel				Higher collagen deposition	
				Elevated CD43 and transforming growth factor beta (TGF-β) expression	
				Decreased DC95+ cells	
**Corneal Injury**	Vvitamin A and cyclosporine A-incorporated into the oily core of protamine nanocapsules	Albino New Zealand rabbits and C57BL/6 mice	Male		^86^
Subjecting the Animal Right Eyes to Prk Surgery with a 2 mm Ablation Zone on the Central Cornea and a Depth of 45 mm					
**Ischemia (Grade 3) and Excisional Wound in the Rabbit Ear (Ear Ulcer Model)**	Administration of S42909, a potent NADPH oxidase inhibitor activity, through self-microemulsion drug delivery system (SMEDDS)	New Zealand white rabbits	Male	Improved wound healing	^87^
				Increased anti-inflammatory cytokines (e.g., TGF-β1 mRNA and IL-10 mRNA at the dose of 100 and 30 mg/kg/d, respectively)	
				Increased collagen deposition and TGF-β1 protein	
				Decreased glycosaminoglycan	
**A Chronic Osteomyelitis**	Composite of vancomycin-loaded *N*-trimethyl chitosan nanoparticles (VCM/TMC NPs) and poly(trimethylene carbonate) (PTMC) “VCM/TMC NP-PTMC”	New Zealand white rabbits	Male	Exhibited excellent antibacterial activity	^88^
				Promoted bone repair	
**Diabetic Model**	Platelet-derived extracellular vesicles (pEVs) together with reduced graphene oxide (rGO)-loaded gelatin-alginate hydrogel (GelAlg) “GelAlg@rGO-pEV hydrogel”	Wistar rats	N/A	Decreased expression of inflammatory biomarkers	^26^
A Full-Thickness (8 mm in Diameter) Using a Punch Biopsy				Regulated immune response	
				Promoted angiogenesis	
				Facilitated diabetic wound healing	
**Diabetic Model (Infected and Non-Infected)**	Composite patch containing, chitosan/collagen/chondroitin sulfate/elastin/hyaluronic acid and loaded with ferumoxytol (as FD-approved superparamagnetic iron oxide nanoparticles (SPION)), follistatin like-1 (FSTL-1) proteins, and AC2-26	Wistar rats	Male	Enhanced healing efficacy, blood vessel formation, and maturation	^28^
A Round Shape (20 mm in Diameter) on the Dorsal Area of Rats Using a Metal Punch				Induced neovascularization	
**Second-Degree Burn Model**	Reconstituted membrane/gellan gum/lysosome RMG–LZ and RMGT–LZ (with and without MSCs)	Wistar *R. norvegicus* rats	Male	Reduced acute/chronic inflammatory infiltrates	^89^
Exposing the Skin to an Iron Bar (Heated at 100 °C)				Increased vascular proliferation and collagen deposition	
				Decreased injury area by 96%, and complete epithelialization after 30 days	
**Burn Model**	NLC-based on olive oil and loaded with eucalyptus oil	Wistar rats	Male	Enhanced healing process	^33^
Three Circular Full-Thickness Burns (4 mm in Diameter) on the Back of the Animals byAluminum Rod (105 °C for 40 s)				Enhanced antimicrobial activity	
A Full-Thickness Excisional Wound (10 mm in Diameter) Using a Punch Biopsy	Topically administered technetium-99 m (^99m^Tc-NLC)	Wistar rats (biodistribution study) CBA/CaOlaHsd mice (sensitization study) New Zealand rabbits (for irritation study)	Female (rats and mic) Male (rabbits)	Exhibited local effect after topical administration with no irritation/corrosion on NLC treated site	^90^
**Diabetic Model**	Sodium carboxymethyl chitosan (NaCMCh)-rhEGF-hydrogel	SD rats	Male	Reduced wound area	^35^
A Wound of (3.14 cm^2^ Circular Area) on the Dorsum of the Rats Using a Round Template					
**Diabetes Model**	Silk fibroin (SF) and poly(lactide-*co*-glycolic acid) (PLGA) hybrid membrane	SD rats	Male	Reduced residual wound area on day 15	^91^
A Wound of (3.14 cm^2^ Circular Area) on the Dorsum of the Rats Using a Round Template	(PLGA/SF (2:1))				
**Diabetic Model**	Polycaprolactone (PCL)-based oxygen-releasing electrospun nanodibers	SD rats	Male	Facilitated vascularization	^48^
A Full-Thickness (2 × 2 cm^2^) Excisional Wound	“Sodium per carbonate (SPC)-loaded PCL”				
**Diabetic Model (Type I)**	A superoxide dismutase (SOD) loaded thermo-sensitive hydrogel-poly(*N*-isopropyl-acrylamide)/poly(γ-glutamic acid) (PP)	SD rats	Male	Enhanced wound closure and healing	^92^
Square Wounds (Side Length 1 cm) on the Left and Right Sides of the Back Spine					
**Diabetic Model**	Black phosphorus (BP)-based gel containing fibrinogen and thrombin	BALB/c mice	Male	Accelerated microcirculatory blood flow and wound healing	^93^
A Full-Thickness Wounds (7 mm in Diameter) Using Scissors				Promoted vascularization and angiogenesis	
				Reduced inflammation	
				Eliminated bacteria	
**Inguinal Hernia Model**	Nanosized fibers decellularized aorta (DA) loaded with autologous bone marrow-derived mesenchymal stem cells	New Zealand white rabbits	Male	No hernia recurrence was observed	^94^
4 cm Inguinal Skin Incision				Enhanced cell infiltration, tissue regeneration, and neovascularization	
**A Surgical Wound**	Cellulose ether-PVA nanofiber mats loaded with halloysite clay (HNT) and gentamicin sulfate (GS)	Wistar rats	Female	Enhanced wound closure rate by 2 weeks in terms of re-epithelialization and enhances collagen deposition	^95^
(25 mm in Diameter) Surgical Scissor	“HNT-incorporated GS-loaded EHEC/PVA electrospun nanofiber mats”			Significant wound reduction	
**Infected Diabetic Wound Model**	Mixture of high-density platinum nanoparticle assemblies (PNAs) with GelMA ± ultrasound (US)	Strain: N/A mice	N/A	Accelerated wound healing	^96^
Full-Thickness Wounds (10 mm in Diameter) Using a Punch Biopsy					
**MRSA-Infected Wounds**	Core–shell Gd-doped Bi_2_S_3_@Cu(II) boron imidazolate framework (Bi_2_S_3_:Gd@Cu-BIF) nanoassemblies	Kunming mice	Female	Promoted cell proliferative activity	^97^
				Enhanced fresh granulation tissue formation	
				Facilitated collagen deposition, and re-epithelialization	
				Accelerated neo-blood vessel formation	
**Non-Infected Wound Model**	VEGF-coated tetrapodal zinc oxide (t-ZnO)-laden GelMA 3D printed hydrogel	SKH-1 hairless mice	Male	Enhanced wound healing	^27^
Two Circle (Bilateral) Defects (9 mm in Diameter) on the Back				Attenuated immunogenicity	
**Non-Infected Wound Model**	LL37, a human antimicrobial peptide, encapsulated in nanostructured lipid carriers (NLCs) NLCs-LL37	db/db (BKS.Cg-m+/+Lepr^db^/J) mice	Male	Improved wound healing	^98^
Two Full-Thickness Wounds (8 mm in Diameter) Using a Punch Biopsy				Enhanced re-epithelization and restoration of the inflammatory process	
**Pressure Ulcer Diabetic (Type I) Model**	Electrospun poly(lactic acid) (PLA)-loaded with calcium-releasing nanoparticles (SG5)	db/db (BKS.Cg- *Dock7* ^*m*^ *+/+ Lepr* ^*db*^ */J*) mice	Male	Facilitated angiogenesis	^99^
				Enhanced collagen deposition and granulation tissue formation	
				Accelerated wound closure	
Two 6.0 mm Diameter Circular Full-Thickness Excisional Wounds	Antagomir-34a in Pluronic F-127 gel (through both topical knockdown and systemic knockdown)	miR-34a knockout mice and C57BL/6J mice	N/A	Both topical/systematic knockdown of miR-4a levels using antagomir gel in KO mice resulted in impaired wound healing	^100^
				Cytokines alteration in miR-34a-deficient wounds (including IL-1β, IL-6, TNF-α, and IL-10)	
**Infected/Non-Infected Burn Injury Wound Model (Second-Degree Burns)**	Electrosup ε-polylysine (εPL) and Dopamine-Loaded Gelatin (Gel) Nanofibers Mats “εPL_Gel_pDA mats”	Juvenile white Yorkshire pigs	Male piglet (noninfected wounds) Female pig (infected wounds)	Reduced hypertrophic scarring	^36^
Direct Contact of Porcine Skin with a Hot Water Beaker (Diameter, ∼4 cm; Surface Area, ∼12.57 cm, 92 °C, for 22 s)				Promoted wound closure and re-epithelialization	
				Antimicrobial activity	
**Diabetic (Type I) Model**	Pt-porphyrin ethylglutamate dendrimer	TallyHo/JngJ mice	N/A	Provided capability to monitor oxygenation changes during the healing process	^101^
A Full-Thickness Wounds (8 mm in Diameter) Using a Punch Biopsy					
**Diabetic Wound Model**	Topical administration of Tat-Rac1 protein	Db/db and wild type (Hemizygous db/wt) mice	Male and Female	Accelerated cutaneous wound healing in both wild type and db/db mice	^102^
Four Full-Thickness Excisional Wounds (6 mm in Diameter)				Enhanced re-epithelialization	
				Reduced inflammation	
				promoted keratinocyte proliferation and migration	
**Diabetic Model**	Combinations of human umbilical cord mesenchymal stem cells (hUCMSC)-derived exosomes “hUCMSC-exos” in Pluronic F-127 (PF-127) gel	SD rats	Male	Accelerated wound closure rate	^103^
Two Full-Thickness Skin Wounds (10 mm in Diameter) with 1.5 cm Between Wounds				Increased expression of CD31 and Ki67	
				Enhanced regeneration of granulation tissue	
				Upregulated expression of VEGF and TGFβ-1	
**Diabetic (Type I) Model**	A self-healing hydrogel composed of carboxymethyl chitosan (CMCS)-protocatechualdehyde (PCA) hydrogel loaded with ultrasmall copper nanoparticles (Cunps) “Cunps@CMCS-PCA Hydrogel”	SD rats	N/A	Enhanced angiogenesis	^49^
A Full-Thickness Wound (2 cm in Diameter)				Improved wound healing	
				Suppressed inflammation	
				Induced macrophage anti-inflammatory transition by deactivating JAK2/STAT3 signaling pathway	
**Rat Liver Trauma Model (Hepatic Hemorrhage Induced with Scissors)**	Pt@MPDA/QX314@Fibrin *in situ* sprayable nanoparticle gel composite	SD rats	Male	Enhanced wound closure.	^104^
Tail Amputation Model (Cut Off 20% of the Tail’s Length)	“Platinum clusters (Pt) loaded-mesoporous polydopamine (MPDA) nanoparticle and QX-314-loaded fibrin gel”			Facilitated collagen deposition	
**Diabetic Model**	Valsartan amphiphile filament hydrogel	Zucker diabetic fatty (ZDF) rat	Male	Promoted wound closure	^105^
A Full-Thickness Skin Wound (1.3 × 1.3 cm)				Downregulation of TGF-β signaling pathway mediators (pSmad2, pSmad3, Smad4)	
				Increased mitochondrial metabolic pathway intermediates	
**Diabetic Wound Model**	A micropatterned nanofibrous scaffold with bioglass (BG) nanoparticles encapsulated inside coaxial fibers (PLLA)	SD rats (there was a confusion in the use of rat and mice in the paper)	N/A	Promoted angiogenesis and collagen deposition	^106^
A Full-Thickness Wound (8 mm in Diameter)	BG@PG			Shortened healing process	
**Excision Wound**	Nanoceria functionalized with folic acid (FA-nanoceria) with unique spray formulation	Wistar rats	N/A	Enhanced wound healing	^107^
A Full-Thickness Skin Wound (2 × 2 cm^2^) on the Dorsal Side				Excellent antioxidant and ROS scavenging capability	

**Table 3:** Distribution of Animal Sexes and Major Outcomes of the Studies Focusing on Biomaterials for Chronic Wound Healing

Wound type	Biomaterial	Animal	Sex	Outcome	Ref
**Excisional Wound Model**	Collagen/chitosan scaffolds bioconjugated with *N*-acetylcysteine (NAC) and ε-poly lysine (ε-PL)	C57BL/6mice	N/A	Enhanced wound healing	^108^
				Reduced lesion size	
				Mitigated inflammation	
**Diabetic/Normal Chronic Wound Model**	“3D SEWH” scaffold, fabricated of egg white (EW) in alkaline solution and cross-linked with Dulbecco’s modified Eagle medium (DMEM)	db/dB mice (diabetic wound healing)	N/A	Enhanced collagen deposition	^109^
A Full-Thickness Wound (10 mm in Diameter) Using a Punch Biopsy		C57BL/6 mice (normal wound healing)	N/A	Enhanced diabetic/normal wound recovery	
**Diabetic (Type I)**	A 3D-printed alginated (ALG)/chondroitin sulfate methacryloyl (CHSMA) patch cross-linked with acrylate-modified VEGF (mVEGF) using UV irradiation	C57BL/6J mice	N/A	Accelerated wound healing	^110^
A Full-Thickness Wound (10 mm in Diameter)	“patch&mVEGF”			Increased collagen deposition	
				Inhibited inflammatory response confirmed through staining for the IL-1β and TNF-α	
				Enhanced angiogenesis determined using staining for CD31 and α-SMA	
				Promoted macrophage polarization	
**Full-Thickness Wound Model**	Sodium Alginate-Gum Arabic hydrogel loaded with recombinant human Mitsugumin 53 (rhMG53)	C57BL/6 mice	Male	Facilitate dermal wound healing	^111^
Two Full-Thickness Wounds (6 mm in Diameter) Using a Punch Biopsy					
CCl**_4_**-Induced Liver Fibrogenesis Model	BIBF1120 (Nintedanib), tyrosine kinase inhibitor, administration	C57BL/6 mice	Male	Attenuated collagen accumulation and HSC activation	^112^
				Ameliorated intrahepatic inflammation and angiogenesis	
**Critical-Sized Calvarial Bone Defects and Diabetic Cutaneous Wound Models**	Hybrid hydrogel consisting of formyl-met-leu-phe (fMLP) and FasL-conjugated silica nanoparticles (SiO_2_-FasL) loaded in pH-responsive hydrogel (chitosan (CS) modified by 4-formylphenylboronic acid (FPBA))	C57BL/6 mice	Female	Enhanced tissue regeneration	^113^
A Full-Thickness Wound (10 mm in Diameter)	(Gel@fMLP/SiO_2_) hydrogel			Promoted macrophage phenotype polarization toward M2	
**Full-Thickness Wound Model**	Fibroin/fibroin supplemented with gelatin microcarriers	C57BL/6N mice	Female	Fully functional recovery without fibrosis	^114^
Deep Skin Wounds (4 mm in Diameter) Using Biopsy Stylet				Increased expression of pro-inflammatory cytokines TNF, IL-6, IL-1β	
				Increased expression of chemokines CXCL1 and CXCL2	
**Full-Thickness Wound Model**	Human embryonic stem cells (hESCs)-endothelial progenitor cells (EPCs) and human cord blood-derived EPCs (hCB-EPCs)	Athymic nude mice	Male	Higher level of VEGF and Ang-1 secreted by hESC-EPCs compared to hCB-EPCs	^115^
A Full-Thickness Wound (12 mm in Diameter) Using a Punch Biopsy				EPC-transplanted wounds exhibited better regeneration	
**A Full-Thickness Wound Model**	Transplantation of the amnionic membrane (AM)-living skin equivalents (LSE) in wound	BALB/cAJc1-nu nude mice	Female	Enhanced graft survival in wound	
A Full-Thickness Wound (1.5 cm × 1.5 cm) Using Scissors				Enhanced vascularization with good morphology	^116^
**Diabetic/Normal Chronic Wound Model**	Endothelial progenitor cells or human induced pluripotent stem cells (hiPSCs) seeded in AHA-RGD hydrogel	Nude mice	Female	Accelerated wound healing and wound closure	^117^
Two Full-Thickness Wounds (6 mm in Diameter) Using a Punch Biopsy				Enhanced granulation layer formation	
				Accelerated neovascularization	
**Infected Diabetic Wound Model**	Oxidized dextran (ODEX), antimicrobial peptide (SWLSKTAKKLFKKIPKKIPKKRFPRPR PWPRPNMI-NH)-modified hyaluronic acid (HA-AMP) and PRP	db/db mice	Male	Accelerated wound healing	^118^
Two Full-Thickness Wounds (8 mm in Diameter)	“ODEX/HA-AMP/PRP”			Increased collagen deposition and angiogenesis, and VEGF production	
				Significant antibacterial activity	
				Inhibited the expression of pro-inflammatory factors (TNF-α, IL-1β, and IL-6)	
				Enhanced anti-inflammatory factors (TGF-β1)	
**Infected Wound Model**	Oxygenation of carboxylated cellulose nanofibrils (CNF)	CD1 mice	Female	The higher the oxidation level,higher the antibacterial activities	^119^
A Full-Thickness Wounds (10 mm in Diameter) at the Neck Region Using a Scalpel				Reduced bacterial survival by 71%, 24 h post treatment	
**Diabetic Wound Model**	Bioceramic particles (Ca_7_P_2_Si_2_O_16_) loaded in poly(caprolactone) (PCL)/gelatin nanofibrous composite scaffold fabricated through coelectrospinning process	C57BL/6 mice	Female	Enhanced angiogenesis, re-epithelialization, and collagen deposition	^120^
A Full-Thickness Wounds (8 mm in Diameter)				Inhibited the inflammatory response	
**Diabetic/Non-Diabetic Wound Model**	Liquid metal (EGaIn)-antibiotic particles (LA)	BALB/C mice	Male	Reduced bacterial count in the wound	^121^
A Full-Thickness Wound (8 mm in Diameter)				Enhanced wound closure	
**Diabetic Wound Model**	Hot spring-mimetic fayalite (Fe_2_SiO_4_, FA) and *N*,*O*-carboxymethyl chitosan (NOCS) hydrogel	C57BL/6 mice	Male	Accelerated wound healing	^122^
A Full-Thickness Wound (10 mm in Diameter)				Enhanced angiogenesis	
**Diabetic Wound Models**	Graphene oxide-chitosan-calcium silicate (GO-CTS-CS) film	C57BL/6 mice	N/A	Accelerated wound healing	^123^
A Skin Wound (10 mm in Diameter)				Exhibited significant photothermal antibacterial and antitumor efficiency	
**Diabetic/Non-Diabetic Wound Model**	Konjac glucomannan (KGM)-modified SiO_2_ nanoparticles (KSiNPs)	C57BL/6J mice	Male	Accelerated wound healing and closure rate	^124^
A Full-Thickness Dorsal Wound (7 mm Circular) Using a Punch				Enhanced angiogenesis, collagen production	
				Decreased inflammation	
				Induced M2 macrophage polarization	
**Diabetic Wound Model**	Transplantation of living micronized amniotic membrane (LMAM) and decellularized micronized amniotic membrane (DMAM) to the wound	C57BL/KsJ, db/db mice	Male	Promoted diabetic wound healing	^125^
Two Full-Thickness Wound (12 mm in Diameter)				Regulated macrophage migration and phenotype switch	
				Increased neovascularization	
**Diabetic Wound Model**	Homodimer of fibroblast growth factor 2 (FGF2) and poly(ethylene glycols)	TallyHo/JngJ mice	Male	Accelerated wound healing	^126^
A Full-Thickness Dorsal Wound (8 mm Circular) Using a Punch	FGF2-PEG2k-FGF2			Enhanced tissue granulation and blood vessel density	
**Full-Thickness Wound Model**	Glycated collagen matrices implantation	BALB/cJ mice	Male	Delayed wound healing response	^127^
A Full-Thickness Dorsal Wound (10 mm Circular)					
**Full-Thickness Wound Model**	Phosphocreatine-grafted methacryloyl chitosan (CSMP) hydrogel	BALB/c mice	Female	Enhanced wound healing	^128^
A Defect (8 mm in Diameter) Using Scissors				Decreased inflammatory factors such as IL-1β, IL-6, and TNF-α	
**Diabetic Wound Model**	Polyethylene glycol diacrylate (PEGDA)-RGD-thiol-coated magnetic particle (TMP)	BALB/c mice	Male	Enhanced wound healing	^129^
				No adverse reaction was observed in major organs	
				Minimized unwanted inflammation	
**Infected Diabetic Wound Model**	Microneedle bandage functionalized with dopamine-coated hybrid nanoparticles containing selenium and chlorin e6 (SeC@PA) and further modified with modified with l-arginine	BALB/c mice	Male	Accelerated wound healing	^130^
				Exhibited anti-inflammatory properties	
				Promoted M2 macrophage phenotype polarization	
**Cutaneous Wound Model**	Nitric oxide (NO)-releasing dressing consists of electrospun poly(e-caprolactone) (PCL)/chitosan (CS)	BALB/c mice	Male	Accelerated wound healing	^131^
Two Symmetrical Full-Thickness Excisional Wounds (6 mm in Diameter) Using a Punch Biopsy	“PCL/CS-NO” dressing			Increased re-epithelialization and granulation formation	
				Enhanced collagen synthesis and organization of epidermal–dermal junction in regenerated tissue	
**Diabetic Wound Model**	Curcumin (Cur) loaded saccharide-peptide based hydrogel consisting of diphenylalanine (FF)/hyaluronic acid (HA)	BALB/c mice	Male	Promoted chronic wound healing	^132^
A Full-Thickness Skin Wound (10 × 10 mm^2^) on the Dorsal	N-FF/HA-Cur hydrogel			Anti-inflammatory properties	
**Cutaneous Excisional Wound Model**	Silk films, lamellar porous silk films, electrospun silk nanofibers biomaterial with epidermal growth factor (EGF) and silver sulfadiazine (either loaded or coated)	BALB/c mice	Female	Reduced scar formation	^133^
Dorsal Surface (8 mm in Diameter) Using a Punch Biopsy				Enhanced re-epithelialization, dermis proliferation	
				Promoted collagen synthesis and epidermal differentiation into hair follicles	
**Infected Diabetic Wound Model**		C57BL/6 mice	Male	A model suitable for testing novel therapies	^134^
Two Symmetrical Full-Thickness (5 mm in Diameter) Using a Punch Biopsy					
**Diabetic Wound Model**	4-Arm polyethylene glycol (4PEG) functionalized with MMP-2 siRNA (4PEG-siRNA)/linear polyethylenimine (LPEI)	C57BL/6 mice	Female	Accelerated wound healing/closure rate	^41^
Burning (9 mm in Diameter) Using a Circular Metal Stick, Followed by Excision of All the Dead Tissue				Reduced MMP-2 expression level	
				Larger amount of cytokeratin expressed in the recovered tissue	
**Diabetic/Non-Diabetic Wound Model**	5-methylpyrrolidinone chitosan (MPC)-based dressings loaded with neurotensin	C57BL/6 mice	Male	MMP-9 reduction in diabetic wound on day 10	^135^
A Full-Thickness Wound (6 mm in Diameter) Using a Punch Biopsy				Increased collagen deposition and fibroblast migration	
**Diabetic Wound Model**	Proanthocyanidins (PC) doping into gelatin/zirconium (Zr^4+^) hydrogel	C57BL/6N mice	Male	Enhanced wound healing and accelerated collagen deposition	^136^
A Full-Thickness Wound (8 mm in Diameter)	PC@Gel-Zr hydrogels				
**Full-Thickness Wound Model**	ROS-scavenging gelatin-hydroxyphenyl propionic (GH)/antioxidant gallic acid-conjugated gelatin (GGA) hydrogels	C57BL/6N mice	Female	Accelerated wound healing	^137^
A Full-Thickness Wound (8 mm in Diameter)	“GH/GGA hydrogels”			Promoted neovascularization and hair follicle formation	
**Delayed Wound Healing Model**	A starPEG-glycosaminoglycan (GAG) heparin hydrogel	C57BL/6 or db/db mice	N/A	Enhanced wound closure	^138^
A Full-Thickness Wound (6 mm in Diameter) Using a Punch Biopsy				Reduced inflammation	
				Promoted vascularization and increased granulation tissue formation	
**Full-Thickness Excisional Wound Model**	Human adipose tissue derived MSCs (AT-MSCs) cultured on silicone coated by plasma polymerization with a thin layer of acrylic acid (ppAAc)	C57BL/6 mice	Female	Accelerated wound healing	^139^
Two Full-Thickness Wounds (6 mm in Diameter) Using a Punch Biopsy				Downregulated TNF-α-dependent inflammation	
				Increased increase M2 macrophage numbers	
				Promoted angiogenesis and formation of granulation tissue	
				Increased myofibroblast differentiation	
**Diabetic Wound Model**	Hyaluronic acid (HA) hydrogel loaded with Ginkgolide B (GB)	C57BL/6 mice	Male	Promoted healing outcome	^38^
Two Full-Thickness Wounds (0.3 cm^2^) Using a Surgical Scissor				Enhanced re-epithelialization and angiogenesis	
				Reduced inflammation	
**Delayed Wound Healing Model**	Human adipose-derived mesenchymal stromal cells (ADSC) embedded in fibrin gel	C57B6 mice	Female	ADSCs delivered in the stiff fibrin gels accelerated wounds healing (more quickly)	^140^
A Full-Thickness Wound (6 mm in Diameter) Using a Punch Biopsy				ADSCs formed vascular tubes	
**Diabetic/Non-Diabetic Wound Model**	*N*-carboxyethyl chitosan/oxidized dextran/hyaluronan *in situ* gelable hydrogel	B6.Cg-m +/+*Lepr^db^*/J, db/db mice	Male	Accelerated wound healing	^37^
A Full-Thickness Excisional Wound (10 mm in Diameter)		C57BL/Ks mice	Male		
**Full-Thickness Wound Model**	Gelatin hydrogel sheets loaded with platelet derived growth factor-BB (PDGF-BB) concentration in cryopreserved hPL group (C-hPL), cryopreserved and lyophilized hPL group (CL-hPL), and the lyophilized and refrigerated hPL group (L-hPL)	C57bl6J/Jcl mice	Male	The wounds almost healed by day 14 (in all groups)	^141^
A Full-Thickness Wound (6 mm in Diameter) Using a Punch Biopsy	Human platelet lysate (hPL)			Confirmed the stability of the growth factors contained in lyophilized hPL at 4 °C for up to 9 months	
**Full-Thickness Wound Model**	Gelatin hydrogel nonwoven fabrics Genocel and Pelnac sheets	C57BL/6JJcl mice	Male	Higher number of newly formed capillaries in the Genocel compared to Pelnac on day 7	^142^
A Full-Thickness Skin Defect (8 mm in Diameter) Using a Punch Biopsy				Higher pan-macrophage number, M2 macrophage number, and M2 ratio in the Pelnac group compared to Genocel	
**Full-Thickness Skin Defect**	Basic fibroblast growth factors (bFGF)-embedded in gelatin sheet	C57BL/6J mice	Male	Enhanced wound healing	^143^
A Bilateral Full-Thickness Wound (6 mm in Diameter) Using a Biopsy Punch				Enhanced collagen maturity and vascularity	
**Full-Thickness Wound Model**	Natural rubber latex (NRL) from rubber tree (*Hevea brasiliensis*) and FrHB1 (F1)-protein	*Mus musculus* Balb/c mice	Male	Increased F1 protein (0.01%) and collagen	^144^
Two Full-Thickness Wounds (5 mm in Diameter) on Dorsal Cervical Region					
**Infected/Non-Infected Wound Model**	Elastomeric poly(L-lactic acid)–poly(citrate siloxane)–curcumin@polydopamine hybrid nanofibrous scaffold	BALB/c nude mice	Female	Enhanced healing in both noninfected and infected wounds	^145^
	“PPCP matrix”			Promoted the adhesion and proliferation of skin cells	
				Exhibited antibacterial properties	
**Excisional Chronic Wound Model**	Bioglass/albumin composite hydrogel	BALB/c nude mice	Female	Accelerated/enhanced wound healing	^50^
A Full-Thickness Excision (10 mm in Diameter) on the Back				Promoted angiogenesis	
				Exhibited good bioactivity *in vivo*	
**Full-Thickness Wound Model in Tumor Bearing Mice**	Spin coating of CaCuSi_4_O_10_ NPs on the surface of poly(ε-caprolactone) and poly(D,L-lactic acid) (PP) electrospun fibers	BALB/c nude mice	Male	Enhanced chronic wound healing	^146^
Subcutaneous Injection of B16f10 Cells Followed by a Full-Thickness Skin Wound (10 mm in Diameter) Directly above the Tumor				Promoted angiogenesis and re-epithelization	
**Infected Wound Model**	Raman tag 3,3′-diethylthiatricarbocyanine iodide (DTTC)-conjugated gold–silver nanoshells + near-infrared laser	BALB/c mice	Female	Enhanced wound healing	^147^
A Circular Skin Wound (7 mm in Diameter) Using a Scalpel	AuAgNSs-DTTC			Demonstrated minimal toxicity in wound bed and collateral damage to vital organs	
**Infected/Non-Infected Wound Model**	Self-assembly of chitosan (CS) and puerarin (PUE) “C@P Hydrogels”	BALB/c mice	Female	Accelerated wound healing	^148^
A Full-Thickness Skin Wound (8 mm in Diameter)				Exhibited a significant antibacterial activity	
				Mitigated inflammation within the wound bed	
**Infected/Non-Infected Wound Model**	Alginate polymers incorporated with growth factor and an array of colorimetric glucose sensors	BALB/c mice	Male and Female	Enhanced wound healing in both infected and noninfected wounds	^73^
Two Full-Thickness Skin Wounds (6 mm in Diameter)				Exhibited wound contraction in both sexes	
				Male mice exhibited higher healing rate and better response to antibiotics	
**Infected Wound Model**	Polymeric bactericidal hydrogel (P-BAC hydrogel) (cross-linking of PVA, *N*-(2-hydroxypropyl)-3-trimethylammo-nium chitosan chloride-AgCl nanocomposite, and proline)	C57/BL6/BALB/C mice	Female	Accelerated wound healing	^149^
Size: Wound (8 mm in Diameter)				Enhanced collagen deposition at wound side	
				Antibacterial properties	
**Infected Wound Model**	Sulfopropyl *p*-aminodiphenylamine (SPA) in a calcium alginate hydrogel preloaded with horseradish peroxidase (HRP) “CA@SPA-HRP hydrogel”	C57BL6/J mice	N/A	Accelerated wound healing	^150^
A Full-Thickness Skin Wound (8 mm in Diameter) on the Back				Exhibited antibacterial properties	
**Colonic Mucosal Wound Model**	Human mesenchymal stem cells (hMSCs) treated with IFN-γ-tethered four-arm maleimide-end functionalized PEG macromer (PEG-4MAL) hydrogels	NOD-SCID IL2Rg-null (NSG) or C57/B6 mice	Male	Accelerated healing of mucosal wound	^151^
An Injury on the Colonic Mucosa at 5 Sites along The Dorsal Artery (1 mm^2^) Using Biopsy				Suppressed activated T cells proliferation and monocyte-derived dendritic cell differentiation	
**Diabetic Wound Model**	Collagen/hyaluronan based hydrogels releasing sulfated hyaluronan “HA-AC/coll hydrogels”	db/db mice	N/A	Accelerated tissue repair, wound healing, and wound closure	^29^
Two Full-Thickness Skin Wounds (6 mm in Diameter) Using a Punch Biopsy				Reduced inflammation	
				Increased vascularization	
**Diabetic Wound Model**	Silk fibroin (SF) and carboxymethyl cellulose (CMC) encapsulated with MnO_2_	N/A	Male	Promoted angiogenesis	^152^
A Full-Thickness Skin Wound (10 mm in Diameter)	SF/CMC@MnO_2_ hydrogel	Mice		Reduced inflammation	
				Balanced the expression of MMPs in diabetic wounds	
				Promoted remodeling of ECM	
**Infected Wound Model**	Polyvinyl alcohol (PVA)/polydopamine (PDA)/Ti_3_C_2_T_*x*_ (MXene)/copper sulfide nanoparticles (CuS) hydrogel+ NIR	N/A	Male	Enhanced skin regeneration ability	^153^
A Full-Thickness Skin Wound (6 mm in Diameter) on the Back		Mice		Promoted capillary angiogenesis and collagen deposition	
				Enhanced antibacterial properties	
**Diabetic And Non-Diabetic Wound Model**	Silk-gold nanorods (GNR) and histamine lasered into wound bed	(BKS.Cg Dock7^m^ +/+ Lepr^db^/J; hereafter db/db) Mice and BALB/c mice	N/A	Enhanced wound healing and accelerated wound closure	^154^
Mid-Dorsal Full-Thickness Wounds (3.5 or 5 mm in Diameter) Using a Punch Biopsy			N/A	Increased transient neoangiogenesis	
				Enhanced biomechanical recovery of skin	
**Infected Diabetic Wound Model**	CuS-quercetin (Qu)–carbonized nanogels (CNGs) nanocomposites + NIR	(BKS.Cg Dock7^m^ +/+ Lepr^db^/J (db/db) mice	Male	Accelerated wound healing	^155^
A Circular Wound (10 mm in Diameter) Using a Surgical Scissors				Suppressed pro-inflammatory cytokines at the wound bed	
				Promoted angiogenesis, epithelialization, and collagen synthesis	
**Infected Wound Model**	MSCs derived from both adipose tissue and hair follicle combined with sponge-like scaffolds (SLS) made of valorized soy protein and β-chitin	(BKS.Cg-m +/+ Lepr^db^/J) db/db mice	Male	Accelerated healing time	^77^
Two Full-Thickness Wound (8 mm in Diameter) Using a Biopsy Punch				Maintained cell viability for several days	
**Diabetic Wound Model**	Deferoxamine (DFO) transdermal drug delivery system (TDDS) using surface microtexturing technology	(BKS.Cg Dock7^m^ +/+ Lepr^db^/J (db/db) mice	Male	Accelerated skin wound healing	^156^
An Excisional Wound (6 mm in Diameter) Using a Biopsy Punch				Increased wound vascularity	
				Promoted collagen deposition and increased dermal thickness	
**Pressure-Induced Ulcer Model in Diabetic Mice**	TDDS containing DFO	(BKS.Cg-m +/+ Lepr^db^/J) C57BL/6 mice	Male	Accelerated wound healing	^157^
**Two Ceramic Magnets (12 mm in Diameter, 5 mm in Thickness with 2.4 g)**				Prevented diabetic ulcer formation	
				Enhanced collagen density	
				Improved neovascularization	
				Reduced free radical formation	
**Chronic Ulcerative Colitis (UC) Model**	Curcumin (CUR) loaded in pluronic F127 (P127)-modified gold shell (AuS)-polymeric core nanotherapeutics	C57BL/6J IL-10–/– BALB/Cmice (Chronic UC)	Male	Mitigated colitis symptoms	^158^
Created via the Addition of DSS (3.5%, W/V) in Drinking Water	P127-AuS@CUR (+transient mild photothermia (TMP))	BALB/c mice (for safety evaluation)	Female	Regulated genes associated with antioxidation, anti-inflammation, and wound healing	
**Diabetic Wound Model**	Syndecan-4 proteoliposomes (S4PL) together with platelet derived growth factor-BB (PDGF-BB)	ob/ob (B6.Cg-Lepob/J)	N/A	Induced alteration in macrophage phenotype with increase in M2 polarization (CD163+)	^159^
Two Full-Thickness Wounds (5 mm in Diameter) Using a Punch Biopsy		Mice		Promoted angiogenesis (increased α-SMA)	
				Enhanced wound healing	
**Diabetic Wound Model**	Alginate disk to deliver syndecan-4 proteoliposomes (“syndesomes”) mixed with FGF-2	ob/ob (B6.Cg-Lepob/J)	N/A	Altered cytokine profile alterations in the wound	^160^
Two Full-Thickness Wounds (5 mm in Diameter) Using a Punch Biopsy		Mice		Promoted macrophage polarization toward the M2 phenotype	
				Enhanced wound healing/closure	
**Infected Wound Model**	Ibuprofen (IBU)-loaded porphyrin-covalent organic framework (COF)-based membrane	White mice	Female	Exhibited excellent antibacterial and anti-inflammatory properties	^161^
Full-Thickness Cutaneous Wound (8 mm in Diameter)	“IBU@DhaTph-membrane”			Enhanced tissue remodeling	
**Full-Thickness Wound Model**	Trilayered nanofibrous scaffold; first layer: (silk fibroin protein blended with polyvinyl alcohol (SF-PVA)); second layer: silk sericin (SS)/PVA loaded with silver(I) sulfadiazine (SSD) (SS/SSD); and third layer: (SF/polycaprolactone (PCL))	BALB/c mice	Male	Improved wound healing	^162^
A Full-Thickness Wounds (5 mm in Diameter) Using a Biopsy Punch				Reduced bacterial infection	
**Infected Wound Model**	Chitosan–indocyanine green (ICG)/luteolin (LUT) nanocomposites	BALB/c mice	Male	Exhibited bacteria-killing	^163^
Two Full-Thickness Wound (5 mm in Diameter)	ICG/LUT-CS + NIR			Eliminated biofilm	
A Subcutaneous Incision Wound (15 mm in Diameter)	Methacrylated hyaluronic acid (MEHA) and maleated hyaluronic acid (MAHA)	BALB/c mice	Male	No significant chronic inflammation reaction observed after implantation	^164^
**A Full-Thickness Wound Model**	Bovine decellularized ECM-derived hydrogel	BALB/c mice	Male	Enhanced wound closure	^165^
Wounds (4 mm in Diameter)					
**Infected Wound Model**	Antimicrobial KK(SLKL)3KK peptide sequence (AMP)–aldehyde hyaluronic acid (HA) hydrogel	BALB/c mice	Female	Significant antimicrobial activity	^166^
A Full-Thickness Wounds (7 mm in Diameter)	“AMP–HA Hydrogel”			Enhanced wound healing	
**A Burn Defect (2°) Model**	Peracetic acid-processed decellularized human amniotic membrane (HAM)	BALB/c mice	Male		^167^
(10 mm in Diameter) Using a Manual Hot Device on the Back					
**Infected Wound and Diabetic Wound**	Prussian blue (PB)@PDA@Ag nanosystem ± NIR	BALB/c mice (for infected wound)	Female	Accelerated infected/diabetic wound healing	^168^
Circular Wounds (∼13 mm in Diameter)		MKR transgenic diabetic mice (for diabetic wound)	Female	Eradicated local inflammatory response	
				Up-regulated VEGF expression	
**Full-Thickness Cutaneous Wound Model**	HAM seeded with mesenchymal stem cell (MSCs) supernatant activated macrophages	BALB/c mice	N/A	Accelerated wound healing	^30^
Two Symmetric Circular Incisions (6 mm in Diameter) Using a Biopsy Punch				Reduced scarring in the wound	
				Decreased inflammatory phase and scar tissue development	
				Increased angiogenesis	
**Infected Wound Model**	Nanoliquid dressing consists of copper sulfide (CuS) + dilute nitric acid (HNO_3_) + NIR	BALB/c mice	Female	Accelerated healing rate	^169^
A Hole (8 mm in Diameter)				Reduced inflammatory response	
**Acute Wound Model and Diabetic Wound Model**	Perfluorodecalin-encapsulated albumin nanoparticles (FDC@HSA), called nano-oxygenated (NOX) powder incorporated in hyaluronate gel (NOX-gel)	BALB/c mice	Male	Accelerated wound healing	^170^
A Round Full-Thickness Wound (15 mm in Radius) Using Ophthalmic Scissors				Exhibited biocompatibility in both wound models	
**Chronic Wound Model**	Bioactive akermanite/alginate composite hydrogel	BALB/c mice	N/A	Accelerated wound healing	^171^
A Full-Thickness Excision (10 mm in Diameter)				Enhanced blood vessel formation and re-epithelialization	
**Chronic Wound Model**	Engineering Mg^2+^-coordinated asiatic acid (AA)-Mg/bacterial cellulose (BC) hydrogel	BALB/c mice	Male	Accelerated wound healing	^172^
A Round Wound (5 mm in Diameter) Using a Biopsy Punch				Promoted collagen deposition, tissue granulation, and revascularization	
**Full-Thickness Wound Model**	Dimethylox-alylglycine (DMOG) and ciprofloxacin (CIP)-loaded poly(3-hydroxybutyrate-*co*-4-hydroxybutyrate) (P34HB) fibrous wound dressing	BALB/c mice	Male	Accelerated wound healing	^173^
A Full-Thickness Excision (10 mm in Diameter)	“P34HB/CIP/DMOG”			Promoted angiogenesis, remodeling, re-epithelialization and collagen formation	
				Reduced inflammatory response	
**Chronic Wound Model**	Sodium alginate (SA)-hardystonite (HS) composite hydrogel	BALB/c nude mice	N/A	Enhanced wound healing	^174^
A Circular Skin Wound (10 mm in Diameter)				Promoted blood vessel and epithelium formation	
				Inhibited bacterial growth	
				Promoted angiogenesis	
**Full-Thickness Wound Model**	Human adipose-derived stem cells (hASCs) laden in gelatin microcryogels	BALB/c nude mice	N/A	Accelerated wound healing	^175^
**Induced Pressure Wound**	Genipin-cross-linked–chitosan hydrogels	BALB/c wild type mice	Female	Enhanced cellular proliferation	^176^
**Diabetic Wound Model**	The laminin-derived dodecapeptide A5G81 immobilized within poly(polyethylene glycol cocitric acid-*co*-*N*-isopropylacrylamide) (PPCN)	(B6.BKS(D)-Lepr^db^/J; (db/db) mice	N/A	Accelerated tissue regeneration and wound healing	^177^
A Full-Thickness Wounds (6 mm Circular) on the Center of Splinted Area					
**Diabetic Wound Model**	Co-delivery of ARA290 (cellular protective peptide) and keratinocyte growth factor (KGF) using elastin-like peptide (ELP)	(BKS.Cg Dock7^m^ +/+ Lepr^db^/J; hereafter db/db) mice	N/A	Accelerated wound healing	^178^
Two Full-Thickness Wounds (10 mm in Diameter) Using a Biopsy Punch	(1:4 KGF-ELP to ARA290-ELP ratio reported to be most effective)			Regenerated epidermis after 28 days	
				Resulted in thicker granulation tissue	
**Chronic Wound Model**	Human keratin matrix (HKM)	(BKS.Cg Dock7^m^ +/+ Lepr^db^/J; hereafter db/db) mice	Female	Accelerated wound closure	^179^
Four Full-Thickness Wounds (6 mm in Diameter) Using a Biopsy Punch					
**Diabetic Wound Model**	QHREDGS (gluta-mine-histidine-arginine-glutamic acid-aspartic acid-glycine-serine) peptide loaded in chitosan–collagen hydrogel	(BKS.Cg Dock7^m^ +/+ Lepr^db^/J; hereafter db/db) mice	Male	Accelerated and enhanced wound closure	^180^
Full-Thickness Middorsal Wound (8 mm in Diameter) Using a Biopsy Punch				Promoted re-epithelialization and formation of granulation tissue	
**Diabetic Wound Model**	Heparin-mimetic peptide (Lauryl-VVAGEGDK (pbs)S–Am (GAG-PA) and Lauryl-VVAGK-Am (K-PA)) amphiphile (PA) nanofiber gel	BKS.Cg-+Lepr^db^/+Lepr^db^/OlaHsd db/db mice	N/A	Enhanced wound healing and neovascularization	^181^
Two Full-Thickness Wounds (6 mm in Diameter) Using a Biopsy Punch				Promoted collagen deposition, angiogenesis, and expression of α-SMA	
				Eliminated TNF-α and IL-6	
**Splinted Excisional Wound Model in Diabetic Mice**	Panthenol citrate (PC) and in the form of poly(panthenol citrate polyethylene glycol citrate co-*N*-isopropylacrylamide) (PC–PPCN)	BKS.Cg-Dock7^m^ +/+ Lepr^db^/J	Male	Enhanced tissue regeneration	^182^
		db/db mice		Improved mechanical strength and electrical properties	
				Reduced inflammation and oxidative stress	
				Promoted re-epithelialization and neovascularization	
**Diabetic Wound Model**	Electrostatically assembled wound dressings as a platform for spatial release of anti-miR-92a, as miR-92a into endothelial cells	(BKS.Cg Dock7^m^ +/+ Lepr^db^/J; hereafter db/db) mice	mix of male and female	Derepresses target transcripts resulted in accelerated wound healing and wound closure	^183^
A Full-Thickness Wound (6 mm in Diameter) Using a Biopsy Punch				Induced a sex-dependent increase in vascularization	
				Promoted angiogenesis	
**Diabetic Wound Model**	Silk fibroin binding peptide (SFBP)-Gluc-MS2 (SGM)-miR146a-Exo@silk fibroin patch (SFP)	BKS-Lepr^em2Cd479^/Nju Db/db	Male	Exhibited less inflammation, collagen deposition, and neovascularization	^184^
A Full-Thickness Wound (10 mm in Diameter) Using a Biopsy Punch	“SGM-miR146a-Exo@SFP”	Mice		Anti-inflammatory and regenerative capability	
**Infected Wound Healing**	Metal-polyphenol polypeptide nanocomposite scaffold (PEAPF) with or without NIR	Kunming	Female	Facilitated skin tissue regeneration	^185^
Two Full-Thickness Skin Wounds (Approximately 8 mm in Diameter)		Mice			
**Infected Diabetic Wound Model**	Chitosan/hyaluronic acid hybrid hydrogel (noncross-linked)	Kunming	Male	Enhanced wound healing	^186^
A Full-Thickness Skin Wound (8 × 8 mm^2^)		Mice		Enhanced antibacterial activity	
				Promoted fibroblast proliferation and migration	
				Enhanced collagen deposition and angiogenesis	
**Diabetic/Non-Diabetic Wound Model**	Human umbilical cord mesenchymal stem cell lyophilized powder (hUC-MSCs) loaded in polysaccharide-based hydrogel consisting of ulvan dialdehyde, chitosan, dopamine (DPA) and silver nanoparticles (Ag NPs)	Kunming	Male	Enhanced diabetic wound healing	^187^
A Full-Thickness Wound (10 mm in Diameter)	“UC-DPA-Ag@hUC-MSCs hydrogel”	Mice			
**Full-Thickness Skin Wound Model**	LVGKLLKGAVGDVCGLLPIC peptide sequence abbreviated as “OM-LV20”	Kunming	Male	Promoted wound healing	^188^
Two Full-Thickness Wound (8 × 8 mm)		Mice			
**Infected Wound Healing Model and Hemorrhaging Liver Model**	HPEM scaffolds containing poly(glycerol-ethylenimine), Ti_3_C_2_T_*x*_ MXene@polydopamine (MXene@PDA) nanosheets and oxidized hyaluronic acid (HCHO)	Kunming mice (to study hemostatic effect)	Female	Accelerated wound closure and wound healing	^189^
Wound Healing Model: A Full-Thickness Cutaneous MRSA-Infected Wound (7 mm in Diameter)		BALB/c mice (for distribution evaluation and toxicity assay)	Female	Promoted cell proliferation and angiogenesis	
				Enhanced collagen deposition and stimulated tissue granulation	
**Infected Wound Model**	Ag_2_S nanodots conjugated Fe-doped bioactive glass nanoparticles (BGN–Fe-Ag2S) into biodegradable PEGDA and AIPH solution	ICR mice	Female	Accelerated wound healing	^190^
A Full-Thickness Skin Wound (8 mm in Diameter)	AIPH/BGN–Fe-Ag_2_S/PEGDA solutions ± laser irradiation				
**Infected/Non-Infected Wound Model**	Arginine end-tagging peptide (Pep 6) with threonine-containing peptide amphiphile (TPA) “Hydrogel-RL”/loaded with etamsylate	ICR mice	N/A	Accelerated wound healing	^191^
A Full-Thickness Skin Wound (10 mm in Diameter)				Antimicrobial properties	
				Promoted re-epithelialization collagen fiber formation/deposition	
**Diabetic/Non-Diabetic Wound Model**	Oxidized carboxymethyl cellulose (OCMC)/gelatin *in situ* gelling hydrogel	C57BL/6 mice (diabetic model) and Wistar albino rats (nondiabetic model)	N/A	Accelerated wound healing in both diabetic and nondiabetic	^192^
A Full-Thickness Skin Wound (8 mm in Diameter)			N/A		
Ischemic Flap Model	Oxygen-releasing antioxidant polyurethane (PUAO)-calcium peroxide (CPO) cryogel scaffold	Swiss albino mice	Female	Prevented tissue necrosis (up to 9 days)	^43^
A U-shaped Skin Flap (30 × 10 mm)				Maintained collagen content and tissue architecture	
**Excisional Wound Model**	Hybrid combination of Frutalin (breadfruit lectin)-polysaccharide galactomannan hydrogel	Swiss albino mice	N/A	Accelerated angiogenesis	^193^
Excisional Wound (10 mm in Diameter)				Promoted fibroblast and keratinocyte proliferation	
**Diabetic Wound Model**	Combination of the extracts of garlic (Ga), turmeric (Tu), and fibroin (Fi)	Swiss albino mice	Female	Wound healed in 12 days	^194^
Two Full-Thickness Skin Wounds (6 mm in Diameter) Using a Biopsy Punch	“Ga+Tu+Fi”			Enhanced the growth of collagen fibers	
				Increased fibroblasts number	
				Mitigated blood vessels inflammation	
An Excision (1 cm) on Dorsal Midline Using a Scalpel	Topical neem, *Azadirachta indica*, hydrogel (N-HG) ± silk fibroin (N-SFB-HG)	Wistar albino rats	Male	Accelerated wound healing with no irritation on skin	^195^
**Diabetic Wound Model**	Carbon nanodots (ND) modified bioactive hydrogel CsADMND) and human amniotic membrane derived stem cell (hAMSC) encapsulated CsADMND termed “CsADMND-SC”	Wistar rats	Male	Stimulated early angiogenesis	^196^
Two Full-Thickness Skin Wounds (20 mm in Diameter)				Accelerated wound closure	
				Promoted collagen deposition, re-epithelialization, and formation of distinct organized dermal epidermal junctions	
**Hernia Defect**	*N*-butyl 2-cyanoacrylate surgical glue (Glubran2) as suture threads	Wistar rats	Male	Exhibited mild inflammatory reaction with a small number of macrophages	^197^
A Round Defect (12 mm in Diameter) Bilaterally				Eliminated apoptotic cells	
				Enhanced vascularized tissue formation around the glue	
Subcutaneous Implantation in Dorsal Region	Diclofenac-linked cotton fibers	Wistar rats	N/A	Exhibited excellent anti-inflammatory properties	^198^
**Acute Wound Model**	Heparinized zinc oxide (ZnO) nanoparticles loaded in chitosan-poly(vinyl alcohol) hydrogel	Wistar rats	Male	Promoted wound closure	^199^
A Full-Thickness Cutaneous Wound (10 mm in Diameter) Using Scalpel				Accelerated angiogenesis and re-epithelialization	
				Decrease collagen deposition	
				Increased antimicrobial properties	
**Acute/Full-Thickness Wound Model**	Mesenchymal stem cells (MSCs) (such as placenta-derived MSCs (PLMSCs) and adipose-derived mesenchymal stem cells (ADMSCs)) seeded on acellular amniotic membrane graft (AAM)	Wistar rats	Male	Accelerated wound closure rate	^200^
A Full-Thickness Excisional Wound (10 mm in Diameter) on the Dorsum	Diosmin (DSM)-loaded nanoemulsion (NE) (F1)/fusidic acid (FA) gel	Wistar albino rats	Female (toxicity test) and Male (wound healing)	Promoted re-epithelialization, angiogenesis, and collagen remodeling	
				PLMSCs exhibited better healing outcomes	
Acute Dermal Toxicity Test and Wound Healing Model	“DSM-NEs (F1)-loaded gel”			Enhanced wound healing and anti-inflammatory activity	^201^
**Diabetic Wound Model**	CMC-agarose hydrogel in poly(3-hydroxybutyrate-*co*-3-hydroxyvalerate) nanofibers	Wistar rats	Male	Accelerated wound healing	^202^
A Full-Thickness Skin Wound (20 mm in Diameter)					
**Diabetic Wound Model**	Copper phosphate (Cu_3_(PO_4_)_2_) nanosheets coordinated with tannic acid (TA) chelated nanozyme TA-Cu NS	Wistar rats	Male	Accelerated wound healing	^203^
A Full-Thickness Wounds (6 mm in Diameter)				Promoted angiogenesis, collagen deposition, and re-epithelialization	
				High antioxidant properties with ROS scavenging ability	
**Infected Diabetic Wound Healing**	Exosome laden OxOBand, an oxygen releasing antioxidant, which composed of polyurethane (PUAO)	Wistar rats	N/A	Accelerated wound closure/healing	^204^
Two Wounds (8 mm in Diameter)	(PUAO–CPO-Exo) cryogel scaffolds			Enhanced collagen deposition, re-epithelialization, angiogenesis, and neo-vascularization	
				Decreased oxidative stress	
Rats: Short Dorsal Skin Incisions (10 mm in Length)		Wistar rats (subcutaneous implantation)	Male	Exhibited the capacity to seal large lung leakage	^205^
Lung: Rat Lung Leakage Model (3 mm in Length; 5 mm in Depth)		Pigs	N/A	Obviated the needs for sutures	
Pigs: A Standardized Visceral Pleural Defect (15 mm in Length; 15 mm in Width; 1 mm in Depth)				Enhanced *in vivo* degradation while maintaining wound healing ability	
**Diabetic Wound Model**	A 3D printed amyloid-based composite hydrogel consisting of bovine serum albumin (BSA) and aloe vera (AV) gel	Wistar	N/A	Accelerated wound healing	^51^
Full-Thickness Open-Excision Wound Using a Biopsy Puncture		Rats		Increased collagen deposition	
				Upregulated VEGF expression and its receptors	
**Deep Second-Degree Burn Wound**	2,3 desulfated heparin (DSH) loaded chitosan microspheres in collagen scaffold (2,3DSH-CM-CS)	Wistar	Female	Accelerated wound healing	^206^
Partial Thickness Wound (10 mm in Diameter and Thickness of 100 mm) Using the Tip of the Rod Soldered to a Circular Iron Disc		Rats		Regulated/mitigated inflammatory events	
**Full-Thickness Abdominal Wall Defect Model**	Tissue based biomaterials: porcine small-intestine submucosa (Surgisis Biodesign; Cook), porcine dermis (Strattice, Firm; patch A, patch D and Permacol), and human dermis (Alloderm) (Graft-JacketTM; Cook)	Wistar rats	Male	Strattice and Alloderm recruited larger early populations of cells than Permacol	^207^
A 30 mm Long Cutaneous Depth Incision					
**Full-Thickness Abdominal Wall Excision Model**	Polypropylene mesh with 2 oligocaprone film and a polydioxanone gluing layer	Wistar rats	Male	Repaired the defects	^208^
	polypropylene mesh, with sodium hyaluronate/carboxy-methylcellulose film.				
Four Skin Longitudinal Incisions at the Back of the Rat and Four Independent Subcutaneous Pockets Made by Blunt Dissection	Chitosan-oxidized hyaluronic acid (HAox) catechol-functionalized terpolymer immersed in FeCl_3_	Albino Wistar rats	Male	Accelerated vascularization	^209^
	“Ch/HAox/T/Fe” interpenetrated polymer network			Promoted the downregulation of IL-1β (pro-inflammatory cytokine)	
Two Parallel Incisions (60 mm Long, 30 mm Apart) Extending Through the Panniculus Carnosus	Positively charged (such as DEAE Sephadex), negatively charged (such as CM Sephadex), or uncharged (such as G-50 Sephadex) beads	Sprague–Dawley (SD) rats	Male	Increase in macrophage was observed in anion exchanger groups	^210^
				Increased in breaking strength in positively charged beads treated groups	
**Full-Thickness Wound Model**	A multifunctional poly(vinyl alcohol)/sodium alginate (SA) “(PVA/SA) hydrogels” embedded with 5-hydroxymethylfurfural (5-HMF) and silver nanoparticles (Ag-NPs)	SD rats	Male	Accelerated wound healing	^44^
A Full-Thickness Wound (25 mm in Diameter)				Reduced inflammation	
				Increased collagen deposition	
				Enhanced angiogenesis and re-epithelialization	
**Infected Diabetic Wound Model**	Cellulose nanocrystals modified with dopamine, cross-linked with gelatin, and further loaded with gentamicin	SD rats	Male	Promoted healing	^211^
A Full-Thickness Wound (6 mm in Diameter)				Enhanced antibacterial performance	
**Infected Diabetic Wound Model**	Poly(ethylene glycol) diacrylate (PEGDA)/catechol-modified hyaluronic acid (HA) hydrogel and Ag-doped mesoporous silica nanoparticle (AMSN)	SD rats	Female	Accelerated wound healing	^212^
A Full-Thickness Wound (7 mm in Diameter) Using a Biopsy Punch	“PEGDA/C-HA-AMSN”			Enhanced antibacterial and anti-inflammatory properties (higher CD31, and lower IL-1β and IL-6 expression)	
**Diabetic Wound Model**	L-nitroarginine (NOArg) based polyester amide (NOArg-PEA) and NOArg-l-Arginine (Arg) co-PEAs	SD rats	N/A	Accelerated wound healing	^213^
Two Full-Thickness Wounds (10 mm in Diameter)				Improved re-epithelialization	
				Increased angiogenesis and collagen deposition	
**Seawater-Immersed Wound Defect Model**	Hyaluronic acid (HA) and quaternized chitosan (QCS) hydrogel	SD rats	Male	Enhanced wound repair	^214^
Four Full-Thickness Wounds (12 mm in Diameter)	“(OHA/HA-ADH/O-HACC and OHA/HA-ADH/N-HACC) composite hydrogels”			Antibacterial properties	
				Decreased pro-inflammatory factors	
				Enhanced anti-inflammatory factors	
**Diabetic Wound Model**	Biocellulose (BC)/Aloe vera gel extract (AE) sheet	SD rats	Male	Promoted wound healing	^215^
The Excision of (1.5 cm × 1.5 cm)				Normal cell arrangement was observed in BC/AE treated animals without fibrosis	
**Diabetic Wound Model**	Human fibroblast growth factor (FGF)-treated adipose-derived stem cells (ADSCs)	SD rats	Male	Accelerated wound healing and revascularization	^216^
A Full-Thickness Wound (6 mm in Diameter)				Enhanced blood flow	
Diabetic Rats and Full-Thickness Excisional Skin Wound Model	Snail glycosaminoglycan (GAG), with bioactive AFG component/methacrylated gelatin (GelMA) double-network hydrogel known as “AFG/GelMA”	SD rats (wound healing/degradation evaluation)	N/A	Attenuated inflammation	^217^
A Full-Thickness Wound (10 mm in Diameter)		BALB/c mice (immunogenicity evaluation)	N/A	Promoted macrophage polarization toward M2 phenotype	
				Inhibited NF-kB signaling pathway	
**Diabetic Wound Model**	Polyurethane-hyaluronic acid (PUHA) hybrid hydrogel scaffolds	SD rats	Male	Accelerated wound healing	^42^
A Full-Thickness Wound (8 mm in Diameter)				Modulated macrophage polarization	
				Downregulation of cytokine-cytokine receptor interaction genes	
Chronically Implanted Shuttles in Brain (Large (300 μm × 125 μm) and Small (100 μm × 125 μm) Size)	Carboxymethylcellulose (CMC) delivery vehicle (shuttle) (dissolvable insertion needle)	SD rats	Male	Exhibited limited secondary damage after implantation (smaller CMC shuttle)	^218^
				Wound closure by 4 weeks	
**Diabetic Wound Model**	Self-Assembled Peptide Hydrogels (RADA16 (Ac-RARADADARARADADA-NH_2_) and RADA16-SP (Ac-RARADADARARADADAGGR PKPQQFFGLM-NH_2_) (mixed at a ratio of 200:7 (v/v)) and conjugated with Substance P	SD rats	N/A	Accelerated wound closure	^219^
A Full-Thickness Wound (10 mm in Diameter)				Enhanced collagen deposition and angiogenesis	
**Full-Thickness Wound Model**	Polycaprolactone (PCL) electrospun nanocomposite loaded with titanium dioxide nanorods (TNR)	SD rats	Male	Accelerated wound healing	^220^
Two Full-Thickness Wounds (1.5 × 1.5 cm)				Promoted cell migration and proliferation	
				Enhanced angiogenesis	
				Enhanced blood vessel formation	
**Infected Wound Model**	Gallium ions (Ga^3+^)-Lactoferrin (LTf) complex	SD rats	Male	Exhibited high antibiofilm activity	^221^
				High biocompatibility without any adverse effects on major organs	
**Full-Thickness Wound Model**	Gelatin-chitosan eggshell membrane (ESM) cross-linked cryogel	SD rats	Female	Accelerated wound healing	^222^
A Full-Thickness Wound (6 mm in Diameter)				Enhanced re-epithelialization	
**Diabetic Wound Model**	Phosphatidylserine (PS)-containing nanoliposomes (D-PSLs and C-PSLs)	SD rats	Male	D-PSLs exhibited better outcomes	^223^
Excisional Wound (15 mm in Diameter)				Accelerated wound closure	
				Increased CD31 expression, vascular endothelial marker	
				Promoted M2-like macrophage polarization	
**Infected Wound Model**	A membrane consists of two layers: (1) poly(lactic-*co*-glycolic) acid (PLGA) and black phosphorus-grafted chitosan (HACC-BP), (2) mixture of gelatin (Gel) and ginsenoside Rg1 (Rg1)	SD rats	N/A	Accelerated wound healing	^224^
Four Full-Thickness Wounds (12 mm in Diameter) by Excising the Dorsum	“Rg1/BP@BM”			Upregulating Ki67, CD31, α-SMA, and TGF-β1	
				Downregulating NF-α, IL-1β, and IL-6	
				Promoted M2 polarization and inhibited M1 polarization of macrophages	
**Acute and Chronic Wound Model**	A polysaccharide based-paramylon hydrogel	SD rats	N/A	Accelerated wound healing	^225^
A Full-Thickness Wounds (8 mm in Diameter) Using a Biopsy Punch				Reduced inflammation	
				Promoted angiogenesis	
				Anti-inflammatory properties with the ability to reduce ROS	
**Full-Thickness Wound Model**	Meropenem-loaded thermos-responsive hydrogels fabricated through the self-assembly of hyaluronic acid and kappa-carrageenan (κ-Carrageenan)	SD rats	Male	Accelerated wound healing and wound closure	^226^
Excisional Wound (1 × 1 cm) Using a Surgical Blade and Forceps					
**Diabetic Wound Model**	Endothelial growth factor (EGF)-loaded in polydopamine modified lyophilized collagen hyaluronic acid scaffold	SD rats	Male	Accelerated wound healing	^227^
A Full-Thickness Skin Excisional Wound (20 mm in Diameter) Using a Biopsy Punch	“CHS-PDA-2@EGF composite scaffold”			Reduced inflammation	
**Infected Diabetic Wound Model**	Chitosan derivative cross-linked by four-armed aldehyde-terminated polyethylene glycol (4-arm PEG-CHO) and covalently bounded to polyaniline (PANI) and deferoxamine (DFO)	SD rats	Male	Enhanced vascularization	^228^
A Full-Thickness Wounds (12 mm in Diameter)	“QP-P-D hydrogels”			Upregulated hypoxia-inducible factor-1α (HIF-1α) expression	
**Infected Diabetic Wound Model**	Polyaniline (PANI) and sulfonated hyaluronic acid (SHA) (a macromolecular dopant)	SD rats	Male	Accelerated infected chronic wound healing	^229^
Four Full-Thickness Wounds (10 mm in Diameter)				Superior antibacterial properties	
**Diabetic Wound Model**	Ferrocene (Fc)-hyaluronic acid (HA) organic copolymer (FHoCP)	SD rats	Male	Accelerated wound healing	^230^
Three Full-Thickness Wounds (8 mm in Diameter)				Reduced inflammation	
				Enhanced angiogenesis and antibacterial effects	
**Infected Wound Model**	Dextran-based hydrogel composed of GelMA and oxidized dextran, and loaded with black phosphorus (BP) nanosheets and zinc oxide (ZnO) NPs	SD rats	Male	Accelerated wound healing/closure	^231^
Four Full-Thickness Wounds (12 mm in Diameter)	“Gel/BP/ZnO + NIR”			Enhanced neovascularization	
				Antibacterial activity	
**Infected Wound Model**	Polydopamine modified poly(ε-caprolactone-*co*-glycolide)-*b*-poly(ethylene glycol)-*b*-poly(ε-caprolactone-*co*-glycolide) (PDA/P2) triblock copolymer and loaded with silver nanoparticles	SD rats	Male	Accelerated wound healing	^232^
Four Full-Thickness Wounds (10 mm in Diameter)	“PCLGA–PEG–PCLGA triblock polymers” and “PDA/P2–4@Ag hydrogel”			Exhibited antibacterial activity	
				Alleviated inflammation	
				Enhanced angiogenesis and collagen deposition	
**Diabetic Wound Model**	Temperature-sensitive self-adaptive CBP/GMs@Cel and INS	SD rats	Male	Promoted wound healing	^233^
Two Full-Thickness Wounds (8 mm in Diameter)	PVA and chitosan grafted with phenylboric acid (CS-BA) by encapsulating insulin (INS) and gelatin microspheres containing celecoxib (GMs@Cel)			Alleviated inflammation	
				Regulated MMP-9 and glucose level within the wound bed	
**Diabetic Wound Model**	Gelatin/PVA scaffold containing curcumin and Lithospermi radix (LR) extract (GC/L/C) scaffold	SD rats	Male	Increased recovery rate	^45^
Four Wounds (15 mm × 15 mm)				Decreased levels of IL-6 and TNF-α	
				Increased expression of transforming growth factor-beta (TGF-β)	
**Diabetic Wound Model**	Sodium alginate (SA)–bioglass (BG)/desferrioxamine (DFO) hydrogels	SD rats	Male	Accelerated diabetic wound healing	^234^
**A Full-Thickness Wound** (20 mm in Diameter)				Promoted hypoxia inducible factor-1α (HIF-1α) and VEGF expression	
				Promoted vascularization	
**Interstitial Cystitis (IC) Inflammatory Model**	Hyaluronic acid (HA)	SD rats	Female	Significantly decreased induced cytokine secretion	^235^
				Increased sulfated glycosamino-glycan (GAG) production without altering tight junction expression	
				Decreased trans-epithelial permeability and IL-6 and IL-8 secretion	
**Full-Thickness Wound Model**	Artemisia argyi extract (AE) loaded composite hydrogel based on (GelMA)/methacrylate hyaluronic acid (HAMA) and mesoporous silica nanoparticle (MSN)	SD rats	Female	Accelerated wound healing	^236^
Four Full-Thickness Wounds (12 mm in Diameter)	“GelMA/HAMA/MSN@AE”			Upregulating the expression of IL-4, TGF-β1, CD31, and α-SMA	
				Downregulated the expression of TNF-α and IFN-γ	
				Promoted M1 to M2 macrophages polarization	
**Infected Wound Model**	Autologous vascularization (AV)-expanded polytetrafluoroethylene (ePTFE) film	SD rats	Female	Enhanced antibacterial properties	^237^
				Inhibited bacterial biofilm formation	
				Reduced the number of bacterial adhesions	
**Diabetic Wound Model**	Vitamin K3 carnosine peptide (VKC)-impregnated silk fibroin electrospun scaffold (SF-VKC)	SD rats	Male	Enhanced wound healing rate	^238^
A Full-Thickness Wound (10 mm in Diameter)				Exhibited antibacterial activity	
**Diabetic Wound Model**	Cell-seeded cryogel/hydrogel glycol chitosan and difunctional polyurethane (DF-PU)	SD rats	Male	Accelerated wound closure	^239^
**A Full-Thickness Wound** (15 mm in Diameter)				Promoted the formation of granulation tissue	
				Completed re-epithelialization	
				Downregulation of pro-inflammatory cytokine expression	
				Increased secretion of SDF-1 and TGFβ-1 cytokines	
**Diabetic Wound Model**	Co-release of nitric oxide (NO) and l-arginine (l-Arg) from methacrylate poly-l-Arg (PAMA) and poly(β-amino ester) (PAMA/PβAE)	SD rats	Male	Accelerated wound healing	^240^
A Hole-Shaped Back Defect (10 mm in Diameter)				Promoted angiogenesis	
**Diabetic Wound Model**	Photothermally responsive hydrogel consisting of copolymerization of acrylic acid (PAA)/methacrylic anhydride-modified gelatin (GelMA)/lipoic acid sodium (LAS) coated copper sulfide nanoparticles (CuS@LAS), abbreviated as “PAG-CuS”	SD or GK rats	N/A	Accelerated wound healing	^241^
A Full-Thickness Square Shape Wound (8 mm in Diameter)				Promoted ECM production, angiogenesis, and re-epithelialization	
				Downregulated MMP-9 expression	
				Reduced ROS level	
**Diabetic Wound Model**	PVA–chitosan (CS)/sodium alginate (SA)–curcumin (PCSA) hydrogels	SD rats	Male	Accelerated wound healing	^46^
Two Full-Thickness Wounds (15 mm in Diameter)				Enhanced angiogenesis and collagen deposition	
				Downregulation of IL-1β and upregulation of CD31 expression	
**Infected Diabetic Wound Model**	Au-epigallocatechin gallate (EGCG) infused into PVA hydrogel followed by NIR irradiation	SD rats	Female	Accelerated diabetic wound healing	^242^
A Full-Thickness Wound (15 mm in Diameter)	“Au-EGCG@H nanocomposite”			Enhanced angiogenesis and cell migration	
				Reduced oxidative stress in cells	
				Restored impaired mitochondrial function	
**Traumatic Tympanic Membrane (TM) Perforations**	Water-soluble chitosan patches (WSCPs)	SD rats	N/A	Enhanced traumatic TM perforations repair	^243^
				Increased collagen density	
**Infected Diabetic Wound Model**	A dual responsive injectable glycopeptide hydrogel based on phenylboronic acid-grafted oxidized dextran and caffeic acid-grafted ε-polylysine	SD rats	Male	Accelerated wound healing	^244^
A Full-Thickness Wound (10 mm in Diameter)	The mangiferin (MF) encapsulated into pH-responsive micelles (MIC) followed by embedding diclofenac sodium (DS) with anti-inflammatory activities and MIC@MF into the hydrogel			Enhanced angiogenesis	
				Demonstrated anti-infection, antioxidation, and anti-inflammation	
**Infected Wound Model**	Strontium (Sr) ions loaded into poly(L-lactic-*co*-caprolactone) (PLCL) and decorated with polydopamine (PDA) and zinc oxide (ZnO)	SD rats	N/A	Accelerated wound healing	^245^
A Full-Thickness Wound (10 mm in Diameter)	“ZnO/PDA@PLCL@Sr nanofilm“			Exhibited antibacterial activities	
				Regulated inflammation	
				Pro-angiogenic capacity	
				Promoted M2 macrophage phenotype polarization	
**Infected Diabetic Wound Model**	PVA–alginate hydrogel loaded with reduced graphene oxide (rGO) and terbium ions (Tb^3+^)	SD rats	Male	Enhanced wound healing	^246^
Four Full-Thickness Wounds (0.8 cm × 0.8 cm)	“(PVA–SA–Tb)”				
**Infected Diabetic Wound Model**	Polypyrrole or Zn-functionalized chitosan molecules cross-linked with poly(vinyl alcohol) flexible bioelectronics	SD rats	Male	Enhanced wound healing and antibacterial properties	^247^
Four Full-Thickness Wounds (1 cm × 1 cm)	“PCPZ hydrogel”				
**Diabetic Wound Model**	Neurotensin (NT)/gelatin micro-spheres (GMs)/silk fibroin (SF) dressings	SD rats	Male	Improved wound healing	^248^
Four Full-Thickness Wounds (15 mm in Diameter) Using a Biopsy Punch				Promoted collagen deposition, tissue granulation, and fibroblast accumulation	
**Infected Wound Model**	Methacrylate-modified silk fibroin (SilMA)/methacrylated hyaluronic acid (HAMA)/Cu-Epigallocatechin-3-gallate (Cu-EGCG) hydrogel	SD rats	Female	Promoted angiogenesis	^249^
Four Round Full-Thickness Wound (12 mm in Diameter)				Regulated inflammation	
**Infected Diabetic Wound Model**	A conductive hydrogel consisting of heparin–polydopamine (Hep-PDA) and reduced graphene oxide (rGO) added to a polyacrylamide (PAM) network	SD rats	Male	Accelerated chronic diabetic wound healing	^250^
Four Square Full-Thickness Wounds (1 cm × 1 cm)	Hep_20_-PDA_0.8_-rGO-PAM hydrogel			Exhibited antibacterial and antioxidant properties	
				Promoted angiogenesis attributed to the constructed hydrogel inherent conductivity	
**Diabetic Wound Model**	Sodium hyaluronate-modified CaO_2_ nanoparticles together with metformin were loaded into the polycaprolactone tips of microneedles (MNs)	SD rats	Male	Promoted wound healing	^251^
Two Full-Thickness Wound (10 mm in Diameter) on Either Side of the Spine				Exhibited antibacterial properties	
				Suppressed inflammation on wound bed	
**Chronic Tympanic Membrane (TM) Perforation**	Epidermal growth factor (EGF)–releasing radially aligned nanofibrous patches (ERA-NFPs)	SD rats	Female	Stimulated the healing of the chronic TM perforations	^252^
**Chronic Tympanic Membrane (TM) Perforation**	EGF-loaded chitosan patch (CPSs) scaffold	SD rats	Female	Enhanced wound healing	^253^
**Creating a Chronic Tympanic Membrane (TM) Perforation Model**	Ventilation tube (VT) insertion used in conjunction with topical application of mitomycin C and dexamethasone (M/D) (VT-M/D)	SD rats	Male	Successful creation of chronic TMP model by VT insertion	^254^
				Increased collagen deposition and macrophage infiltration	
**Infected Diabetic Wound Model**	Conductive hydrogel consisting of copolymerization of *N*-acryloyl glycinamide (NAGA) with quaternized chitosan-*g*-polyaniline (QCSP) “QCSP-*g*-PNAGA”	SD rats	Male	Promoted wound healing	^255^
Four Full-Thickness Wounds (8 mm × 8 mm)				Exhibited significant antimicrobial activity	
**Diabetic Wound Model**	Magnesium ion (Mg^2+^)-chelated electrospun membranes	SD rats	N/A	Accelerated wound healing	^256^
A Full-Thickness Wounds (10 mm in Diameter) Using a Scalpel				Reduced early wound inflammation	
**Cutaneous Wound Healing**	hUCMSCs encapsulated in a thermo-sensitive hydrogel consisting of (chitosan (CS)/glycerol phosphate sodium (GP)/cellulose nanocrystals (CNC)	Specific-pathogen-free (SPF) SD rats	N/A	Accelerated wound closure	^257^
Four Round Shape Incisions (8 mm in Diameter)	hUCMSCs-(CS/GP/CNC) hydrogel			Promoted collagen deposition, tissue remodeling, re-epithelialization, and hair follicle regeneration	
				Enhanced keratinocyte mature marker expression (K1)	
				Decreased expression of TNF-α and IL-1β (inflammatory factors)	
**Chronic Ventral Hernia Model**	Amniotic fluid allograft (AFA) treatment and porcine acellular dermal matrix (ADM)	Lewis rats	N/A	Enhanced vascularization	^258^
				Upregulated expression of pro-regeneration genes	
				Upregulated M2 macrophage phenotype polarization	
**Chronic Tendon Tear Defect**	TGF−β3-loaded electrospun chitosan coated polycaprolacton (CS–g–PCL) fiber scaffolds	Lewis rats	Male	Resulted in more robust constructs	^259^
	“TGF−β3–CS–g–PCL”			Exhibited minor fibrosis and scar formation	
**Chronic Ventral Hernia Model**	Platelet-rich plasma (PRP) in Porcine cross-linked (cADM) and non-cross-linked ADMs (ncADM)	Lewis rats	Male	Induced neovascularization (higher in PRP-treated ncADMs compared to cADMs)	^260^
A Full-Thickness Abdominal Middle Defect (2 × 3 cm)					
**Full-Thickness Wound Model**	Sandwich-type Fiber Scaffolds (3 layers): with radially aligned	Lewis rats	Male	Promoted re-epithelialization	^261^
A Full-Thickness Excision (20 mm in Diameter) Using a Punch Biopsy	nanofibers at the bottom, square arrayed microwells and nanostructured cues at the top, and microskin tissues in between			Exhibited capability to drain exudate from wound	
**Bacteria-Infected Wound**	Molybdenum disulfide nanosheets (MoS_2_ NSs) with triple enzyme-like activities loaded onto carbon nanotubes (CNTs) folloerd by incorporation into multifunctional hydrogels	N/A	N/A		^262^
A Full-Thickness Wound (8 mm in Diameter)		Rats			
**Infected Diabetic Wound Model**	Silver-loaded polydopamine nanoparticles (PDA@Ag NPs) and recombinant human collagen type III (rhCol III)-encapsulated hydrogel	N/A	N/A	Accelerated wound healing	^263^
Four Full-Thickness Wounds (10 mm in Diameter) Using a Medical Clamp		Rats		Enhanced collagen deposition, cell proliferation, and angiogenesis	
				Exhibited great antibacterial properties	
**Diabetic Wound Model**	(PDA@Ag and Cur NPs/VEGF hydrogel)	N/A	N/A	Eliminated bacteria	^264^
		Rats		Promoted angiogenesis	
				Accelerated wound closure	
**Full-Thickness Wound Model**	Polyglycerol sebacate/polycaprolactone (PGS/PCL) loaded with curcumin/ciprofloxacin (CUR/CIP)/and simvastatin/ciprofloxacin (SIM/CIP)	N/A	Female	Achieved almost complete wound closure after 14 days (only in SIM/CIP group)	^265^
A Full-Thickness Wound (1 × 1 cm^2^)		Rats		Enhanced collagen deposition and angiogenesis in SIM/CIP treated groups	
**Adipose Tissue Defect Repair and Hindlimb Injection Study**	Mannitol microparticles with poly(*N*-isopropylacrylamide (NIPAAm)-*co*-vinylpyrrolidone (VP)-*co*-methacrylate-polylactide (MAPLA))	Lewis rats (hind limb injection studies)	Female	Accelerated cellular infiltration in hindlimb muscle injection	^266^
A Defect (1 × 1 cm) in the Center of the Adipose Tissues		New Zealand rabbits (adipose tissue defect repair)	Female	Enhanced wound healing outcome in rabbit adipose tissue (with negligible inflammation and fibrosis)	
**Vocal Fold Injury/Chronic Scarring**	VF-ECM injectable gel	New Zealand white rabbits	Female	Decreased collagen density and tissue contraction of the lamina propria	^267^
1 mm Up-Angled Microcup Forceps				Evoked minimal humoral immune response	
**Full-Thickness Wound Model**	Chitosan (CS) and chondroitin sulfate (ChS) aerogels	New Zealand rabbits	Male	Enhanced wound closure	^268^
Two Full-Thickness Wounds (0.8 cm^2^) Using a Punch Biopsy				Improved tissue granulation	
**Hyperglycemic Rabbit Ear Ulcer Model**	Co delivery of Rab18 and nitric oxide synthase-3 (eNOS) via fibrin-in-fibrin delivery system	New Zealand rabbits	N/A	Enhanced wound closure	^269^
Four Wounds (6 mm in Diameter) in Each Ear Using Punch Biopsies				Increase angiogenesis	
				Reduced inflammation	
**Third-Degree Burn Wound Model**	Poly(l-Lactide-*co*-Glycolide-*co*-Caprolactone) (PLGC), human clinical-grade fibrin (FIB), and hyaluronic acid (HA) terpolymer scaffold	New Zealand white rabbits	N/A	Promoted mature and scar-less epidermis–dermis formation	^270^
Heat-Induced Third-Degree (Full-Thickness) Burn Wounds Using a Custom-Made Device (4 × 4 cm^2^) Preheated	“PLGCFIBHA”				
Brass Plate Template					
**Chronic Rotator Cuff Tear**	A triple layer bioactive scaffold consisting of Polylactic-*co*-glycolic acid microspheres loaded with connective tissue growth factor (CTGF), transforming GF beta 3 (TGF-b3), BMP2 and embedded in polycaprolactone	New Zealand white rabbits	Male	Enhanced chronic rotator cuff repair	^271^
				Maintained tenogenic function of CTGF	
				Increased aggrecan^+^/collagen II^+^ fibrocartilaginous matrix formation	
				Improved collagen fiber density and continuity	
**Chronic and Acute Tendon Injury Wound Model**	PH/GMs@bFGF&PDA hydrogel consisting of polyvinyl alcohol and hyaluronic acid grafted with phenylboronic acid (BA-HA) by loading polydopamine and gelatin microspheres impregnated with basic fibroblast growth factor (GMs@bFGF)	New Zealand white rabbits	Male	Accelerated wound healing	^272^
Acute Model: a Parallel Incision along the Achilles Tendon with Cutting 2/3 of the Achilles Tendon				Promoted collagen I secretion	
Chronic Model: 2 mm Total Tendon Defect Using a Surgical Drill				Mitigated inflammation	
**Diabetic/Non-Diabetic Wound Healing Around Implant Device**	Bone-anchored percutaneous titanium implant with a thin magnetron-sputtered calcium-phosphate coating on the enossal part of half of the implants	New Zealand white rabbits	Female	Lower density of cortical bone around the implants in diabetic animals	^273^
				No adverse effect on the clinical performance of the percutaneous devices was observed	
**Infected Wound Model**	Carboxymethyl chitosan nanoparticles (CMCNPs) combined with ammonium methylbenzene blue (MB)	Japanese big ear rabbit	N/A	Enhanced wound healing	^274^
				Exhibited efficient bactericidal and biofilm eradication properties when irradiated by a 650 nm laser	
	“CMC–MB NPs”			Reduced bacterial infections	
				Suppressed inflammation	
**Cutaneous Partial-Thickness Wounds**	*In situ* photopolymerizable semi-interpenetrating network (sIPN) and Xeroform	Yucatan pig	N/A	Demonstrated the relationship between the expression of inflammatory mediators and the time course of cutaneous healing	^275^
Wounds Covering 5.4% (300 cm^2^)					
**Diabetic Wound Model**	Textile-star poly(ethylene glycol)-glycosaminoglycan (GAG) composite hydrogel-based wound contact layer (WCL) treatment	Danish X Large White Crossbred	Female	Accelerated wound healing	^276^
Full-Thickness Wounds (2 × 2 cm)		Pig		Promoted angiogenesis, granulation formation, and deposition of collagen fibers	
**Acute Surgical Wound**	Stromal cell-derived factor-1 (SDF-1) protein loaded in alginate scaffolds patch	Yorkshire pigs	N/A	Accelerated wound healing and closure	^277^
12 Full-Thickness Incisions (50 mm Each)				Complete wound healing by day 9	
**Full-Thickness Wound Model**	FastSkin	Pig	Female	Accelerated re-epithelialization	^278^
Six Full-Thickness Skin Wounds (4.2 cm × 4.2 cm) with 800 μm Deep				Enhanced collagen organization	
**Delayed Wound Healing Model**		Yorkshire pig	Female		^279^
Four Full-Thickness Skin Wounds (2.5 cm × 2.5 cm/1.75 cm × 1.75 cm)					
**Full-Thickness Wound Model**	An inorganic orthosilicic acid-releasing spun fiber fleece (SIFIB)	Göttingen mini-pigs	Male	Exhibited great anti-inflammatory properties	^280^
Full-Thickness Wounds (4 × 4 cm) with the Depth of 35 mm				Inhibited the expression and activity of NF-kB	
**A Full-Thickness Wound-Healing Model**	Recombinant human collagen III (rhCol-III) gel (either acellulat or with autologous keratinocytes/with fibroblasts-keratinocyte mixture	Landrace pigs	Female	Enhanced early granulation tissue formation	^281^
14 Deep Dermal Wounds (8 mm in Diameter) Using Punch Biopsy				Gel with cells were totally removed from the wound, while the gel without cells remained intact	
**Full-Thickness Surgical Wound**	Sodium percarbonate (SPO)/calcium peroxide (CPO) *in situ* oxygen releasing dressing	N/A	N/A	Accelerated dermal wound healing	^282^
Four Full-Thickness Surgical Wounds (10 × 10 cm^2^)		Pigs		Increased neovascularization (confirmed using Von Willebrand factor (VWF) and CD31 staining)	
**Burn Model and Excisional Model**	Allevyn or melolin dressing	Yorkshire pigs	Female		^283^
14 Full Thickness Surgical Wounds (3 × 3 cm^2^) with 2.7 mm Depth Using an Electrical Dermatome					

This pattern highlights a pressing need for the field to prioritize the inclusion of both sexes in preclinical studies that evaluate the safety and efficacy of nanomedicine products and biomaterials used for wound healing. Responding to biological sex differences is essential because males and females can exhibit markedly different responses to nanomedicines, driven by hormonal regulation, genetic factors, and physiological variations.^66,71,72,74,75^ These differences can influence nanomedicine functionality, immune response, protein corona composition, and overall therapeutic outcome. By systematically incorporating both sexes into experimental designs, researchers can identify sex-specific responses, uncover underlying mechanisms, and ultimately develop safer, more effective biomaterials tailored to the unique biological profiles of each sex.

This emphasis on inclusive research aligns closely with the broader goals of personalized medicine, which aim to optimize treatment efficacy and minimize adverse effects across diverse patient populations. Despite increasing recognition by funding agencies and regulatory bodies of the importance of considering sex as a biological variable, many studies in the field of chronic wound healing that use nanomedicine and bioengineered therapies continue to overlook this critical factor. The consequence of this oversight is significant: it hampers the development of truly personalized, equitable treatments, potentially reduces overall efficacy, and may contribute to higher recurrence rates of chronic wounds in both men and women. Addressing this gap is imperative to advancing precision nanomedicine and ensuring that therapeutic innovations benefit all individuals, regardless of sex.

## Outlook and Future Directions

Our analysis of the literature revealed a significant gap in the inclusion and analysis of sex as a biological variable in preclinical and clinical research on nanomedicine and regenerative therapies for chronic wounds. Despite this, most studies continue to utilize one sex exclusively, often without direct comparison or consideration of how sex influences therapeutic outcomes, despite growing awareness of sex-specific tissue properties, immune responses, and healing dynamics. This under-representation hampers the development of personalized, effective treatments and may lead to suboptimal efficacy, higher recurrence rates, and unanticipated adverse effects in underrepresented populations.

To address these critical gaps, future research must prioritize the systematic inclusion of both male and female models in both preclinical and clinical studies. Specifically, investigations should go beyond mere inclusion; direct, side-by-side comparisons of sex-specific responses are essential for elucidating underlying biological differences that could influence therapy design and application. Standardizing such approaches strengthens preclinical findings and clinical trial designs, ultimately supporting regulatory policies that mandate sex-based analyses. Additionally, funding agencies and journal peer review processes should incentivize and enforce the integration of sex as a key variable throughout all stages of research. Developing guidelines and best practices for reporting sex-specific data will promote transparency and reproducibility.

Furthermore, advancing personalized medicine in wound care requires the integration of multiomics analyses, such as genomics, proteomics, and immune profiling, with sex-disaggregated data to uncover new biomarkers and therapeutic targets.^66^ This holistic approach can pave the way for tailored interventions that consider patients’ sex, systemic health, and microbiome status.

Finally, the translational implications of incorporating sex-specific nanomedicine and biomaterial products are profound. Designing therapeutics that account for sex-based differences can substantially enhance their safety and efficacy profiles, reducing the risk of adverse events and increasing the likelihood of successful clinical translation. Personalized biomaterials optimized for male or female biological milieus could improve wound healing rates, minimize immune rejection, and address sex-specific pathophysiological factors. Furthermore, sex-aware nanomedicine can enable more precise targeting, controlled release, and improved tissue integration, leading to treatments that are more effective and equitable. Embracing these strategies will be essential for moving nanomedical innovations from experimental settings into routine clinical practice, ultimately contributing to precision medicine and reducing healthcare disparities.

In conclusion, fostering a research landscape that systematically incorporates and analyzes sex differences will be instrumental in the optimization of nanomedical and regenerative therapies for chronic wounds. Such efforts will ultimately improve healing outcomes, reduce disparities, and contribute to more equitable healthcare.
